# Measurement properties of the Child Behavior Checklist-10: an ultra-brief screening updated in longitudinal cohort

**DOI:** 10.3389/fpsyt.2026.1767665

**Published:** 2026-04-30

**Authors:** Chen Jiang, Xuejun Gu, Sixuan Li, Xudong Zhao, Fang Cai, Yi Zheng, Kun Zhang, Linlin Ding, Sujun Hu, Tingting Zhou, Yingbo Lv, Chanrong Fu, Wei Cheng

**Affiliations:** 1 Women and Children’s Hospital of Ningbo University, Ningbo, Zhejiang, China; 2 Ningbo Municipal Center for Disease Control and Prevention, Ningbo, Zhejiang, China

**Keywords:** Child Behavior Checklist, confirmatory factor analysis, exploratory graph analysis, Future of Families and Child Wellbeing Study, measurement invariances, receiver operating characteristic

## Abstract

**Objective:**

To develop a brief behavioral screening tool for youth, we sought to reduce the Child Behavior Checklist (CBCL) by selecting the most informative items, establish its structural model, and evaluate comprehensive psychometric properties for potential attention deficit and hyperactivity disorder (ADHD) screening across diverse populations.

**Methods:**

Longitudinal data from the Future of Families and Child Wellbeing Study (FFCWS) were analyzed across three time points. Using a split-sample approach, we aimed to reduce the 34-item CBCL by integrating exploratory graph analysis (EGA) and graded response model (GRM). The final instrument would be evaluated for measurement properties.

**Results:**

Using a sample of 1,786 youth (49.5% female; 51.4% Black/African American), we systematically reduced the 34-item CBCL to a 10-item version (CBCL-10) with a three-factor structure. The CBCL-10 delineated excellent structural validity, supportive measurement invariances, and high internal consistency. The receiver operating characteristic analysis revealed good diagnostic efficiency for ADHD (area under the curve [AUC; 95% CI] = 0.843 [0.818–0.867]), with optimal sensitivity of 0.797 and specificity of 0.736 at a cutoff score of 3.

**Conclusions:**

The present study successfully developed a psychometrically sound CBCL-10 with a diagnostic efficiency for measuring youth attention/behavioral problems. Without sacrificing measurement quality, CBCL-10 offers clinicians and researchers a feasible assessment tool that captures the most diagnostically relevant attention and behavioral manifestations of ADHD.

## 1 Introduction

Attention deficit/hyperactivity disorder (ADHD), characterized by developmentally inappropriate patterns of inattention, hyperactivity, and impulsivity, is a childhood-onset neurodevelopmental disorder with difficulties often persisting into adulthood (1, 2). As one of the most common neurodevelopmental disorders, ADHD contains a consistent estimate with worldwide prevalence of approximately 5%–7% in children and adolescents and approximately 2.5% in adults (3, 4). A marked gender disparity was found on ADHD, with boys being diagnosed two to three times more than girls in community samples and nearly seven to eight times more in clinical settings (1). ADHD represents a significant public health concern due to its substantial negative effects on overall functioning from the early period to the entire lifespan, including increased risks for academic underachievement, employment difficulties, social problems, psychiatric disorders, substance abuse, and even premature mortality (5, 6). The economic burden of ADHD is also considerable. Annual costs in the United States alone are estimated to be approximately $122.8 billion with ADHD or approximately $14,000 per adult with ADHD (7). Early detection of possible ADHD symptoms enhances treatment and primary prevention in time. Therefore, to assess, diagnose, and screen ADHD, precise measurements on it have been considerably developed since last century.

Several well-established instruments have been developed for ADHD early detection, including the Vanderbilt ADHD Diagnostic Rating Scale (55 items), the Conners Rating Scales (90+ items), and the Swanson, Nolan, and Pelham Questionnaire (SNAP-IV, 18 items) (8–10). These instruments have demonstrated adequate psychometric properties and are widely used in clinical settings. However, they are specific measures that assess only core DSM-based symptom dimensions (i.e., inattention and hyperactivity–impulsivity) without simultaneously capturing broader behavioral manifestations—such as oppositional, threatening, or conduct-related behaviors—that frequently co-occur with and complicate presentation (1, 2, 5, 6). These theoretical distinctions between core ADHD symptoms and frequently co-occurring problem behaviors are well-established in clinical literature, with comorbidity rates of 40%–60% for oppositional defiant disorder (ODD) and 20%–50% for conduct disorder (CD) documented across studies (11, 12). A comprehensive ADHD screening tool should therefore be able to capture both to support differential diagnosis and treatment planning.

The Child Behavior Checklist (CBCL) is a widely used standardized instrument for assessing behavioral problems, emotional difficulties, and social competence in children aged 1.5–5 years and adolescents aged 6–18 years (13). The original CBCL consists of 113 items rated on a three-point Likert scale (0 = not true, 1 = somewhat true, and 2 = very true) based on the frequency of parent/teacher’s recall of children’s/adolescents’ behavior in the past six months. The structure of the original CBCL is an eight-factor model for problematic syndromes: anxious/depressed, withdrawn, somatic complaints, social problems, thought problems, attention problems, rule-breaking, and aggressive behavior (13). Later in 2003, an updated CBCL model was developed to align with DSM-V diagnostic criteria: depressive problems, anxiety problems, autism spectrum problems, attention deficit/hyperactivity problems, and oppositional defiant problems (14). The CBCL has been translated into 85 languages and is administered in more than 35 cultures, making it an internationally validated instrument for its adequate internal consistency, cross-informant equivalence, and validity across multiple cultures and contexts (15–17). In contrast with previously mentioned measures, the CBCL adopts a comprehensive, empirically based approach covering a wider behavioral spectrum, which allows concurrent evaluation of attention problems alongside behaviors (8). The Future of Families and Child Wellbeing Study (FFCWS), a nationwide birth cohort, incorporated a 34-item version of the CBCL across the year-5, year-9, and year-15 waves, providing a unique opportunity to examine behavioral assessment across three key developmental stages within the same longitudinal cohort.

Nevertheless, both the original 113-item or the FFCWS 34-item versions impose substantial respondent burden. A 19-item abbreviated version of the CBCL renamed Brief Problem Monitor (BPM), evaluated by applying item response theory (IRT) and factor analysis, was also proposed to enable a quicker assessment across three aspects: internalizing, externalizing, and attention problems (18, 19). The BPM maintains good psychometric properties while allowing for efficient monitoring of children’s behaviors, yet the BPM has not undergone comprehensive measurement invariance testing across groups or time, limiting confidence in cross-group and longitudinal comparisons (18, 19). Moreover, the BPM was not designed to serve as a targeted tool for core screening. Its six-item attention problem subscale covers only inattention-related behaviors without capturing the broader behavioral manifestations. Its performance on quick clinical screening and detailed behavior measurement thus remains to be explored.

Despite its widespread use, several significant gaps remain in CBCL’s application for ADHD assessment. First, there has been no systematic attempt to develop a quick screening version specifically targeting not only attention problem but also co-occurring problem behaviors, which would enhance clinical efficiency and reduce assessment burden (20). Research consistently demonstrates that a high prevalence of youth with ADHD also meet the criteria for ODD or CD, underscoring the clinical importance of capturing these co-occurring problems within a comprehensive screening framework (11, 12, 21). Second, no stable factorial solution of the CBCL has been established, limiting the measure’s structural validity and theoretical foundation (22). Although the eight-syndrome structure of the full CBCL has been validated in 30 societies, abbreviated versions have shown inconsistent factor solutions across samples (15)—for instance, validation of a Chinese CBCL short form yielded a CFI of only 0.734, way below acceptable thresholds. Furthermore, factor structures have varied across age and sex subgroups, with one-factor versus two-factor solutions emerging differently for boys versus girls and for younger versus older children (23). These structural inconsistencies raise concerns about whether existing abbreviated forms adequately capture the intended constructs across diverse populations. Third, very few studies have conducted comprehensive assessments of measurement invariance across different groups (e.g., age, gender, race, and clinical vs. non-clinical samples), which is essential given documented sex differences in prevalence and symptom presentation, racial/ethnic disparities in diagnostic rates, and developmental fluctuations in symptom manifestation from childhood to adolescence (1, 24). Measurement invariance is essential for meaningful comparisons across groups and over time; without establishing that an instrument measures the same construct in the same way across populations, observed score differences may reflect measurement bias rather than true group differences. Lastly, neither the full CBCL nor its abbreviated versions have been subjected to systematic item reduction using contemporary psychometric approaches, such as combining network psychometrics with IRT framework. The integration of network analysis and IRT offers a dual-method framework for item reduction that ensures both structural coherence and individual measurement quality. These gaps highlight the need for further refinement and validation of the CBCL for ADHD assessment.

Based on the limitations in current CBCL applications for ADHD assessment, this study aims to: 1) develop a brief version of the CBCL for efficient assessing of attention and behavioral problems in youth, capturing both core attention deficits and co-occurring problem behaviors, thereby reducing assessment burden (approximately 1 to 2 min of administration time) while maintaining clinical utility; 2) establish a stable structural solution of the revised CBCL for ADHD assessment that offers updated structural validity; 3) evaluate comprehensive psychometric properties of the proposed brief screening tool, with particular emphasis on examining measurement invariance across multiple groups (e.g., age, gender, race, and clinical status) to ensure that the instrument functions consistently and fairly across diverse populations.

## 2 Materials and methods

### 2.1 Data origin and participants

The datasets from the Future of Families and Child Wellbeing Study (FFCWS) were included as the target database for analyzing the CBCL (25). FFCWS is a nationwide, multi-center, longitudinal cohort spanning from 1998 to 2024 and launched by Princeton University. A multi-stage stratified random sampling method was applied by the FFCWS survey team in all US cities over 200,000 to ensure population representativeness (25). All participants in FFCWS have consented to have de-identified data used for scientific research. The Institutional Review Board of Princeton University has reviewed and approved the initial and follow-up study procedures (approval ID: #5767 and #8061). All datasets were accessed and downloaded by the author team from the FFCWS official website (retrieved from https://ffcws.princeton.edu/).

The records from baseline (T0), year 5 (T1), year 9 (T2), and year 15 (T3) were chosen to accommodate the application of the full 34-item CBCL pool based on the FFCWS survey design (25). The same youth were assessed longitudinally across T1, T2, and T3 within the FFCWS longitudinal framework, constituting a repeated-measures design. We extracted variables from primary caregiver (PCGs) and their offspring, respectively: 1) completed CBCL items by PCGs, PCG race, and PCG age at giving birth and 2) youth age (T3), youth bio-gender, youth race, and youth ADHD diagnosis. ADHD diagnostic status was ascertained through the PCG interview at year 15 (T3), in which PCGs were asked whether a licensed physician or clinical professional had formally diagnosed the youth with ADHD (“Yes” for ADHD group, “No” for non-ADHD group). This parent-reported clinical diagnosis approach is consistent with established epidemiological approaches in large population-based studies (26, 27). The T3 time point was selected as this adolescent assessment is likely to capture more stable diagnostic classifications compared with early childhood assessments (25–27). After loading all sets and variables, records were excluded as some significant variables (e.g., CBCL items) contain missing values, resulting in a final analytic sample with complete longitudinal data across all three waves (**eFigure 1 of Supplementary 1**). This longitudinal structure was preserved throughout all analyses, including the evaluation of time measurement invariance. The collected data on the youth’s status reported by themselves and PCGs were the main direction of the study.

### 2.2 Child Behavior Checklist

The FFCWS includes a subset of the items from the complete 113-item CBCL (25, 28). The 34-item Child Behavior Checklist (CBCL-34) across T1 to T3 was the core measurement from the initial phase. CBCL-34 items were read to each surveyed PCG to indicate whether the statement was not true (0), sometimes/somewhat true (1), or very true/often true (2) of her/his offspring (youth). This 34-item pool is not a separately published abbreviated version, but the items were rather selected by the FFCWS research team to balance comprehensive behavioral assessment with respondent burden. The final reduced-item CBCL was the core measurement at the subsequent phase to evaluate its measurement properties.

### 2.3 Analytic plan

#### 2.3.1 Overview

We used R (version 4.3.1) and corresponding compiler RStudio (version 2022.12.0) to perform all data analyses (29–35). The whole process was mainly twofold: instrument forming and instrument assessing (**eFigure 1 in Supplementary 1**).

At the instrument forming stage, the dataset was 1:1 randomly split into equal halves for the exploratory and confirmatory assessments (36–39). The exploratory assessment mainly aims to iteratively reduce items from the CBCL-34 with multiple rounds. Removal protocol was deployed using exploratory graph analysis (EGA) and graded response model (GRM) for the standardized phases we built and described in **eTable 1 of Supplementary 2**. Item reduction would be terminated once the item removal criteria were not met. All possible structural models of exploratory steps and previous conceptual settings would be compared to determine the final version and structure.

At the instrument assessing stage, the measurement properties were evaluated covering structural validity, measurement invariance, internal consistency, and criterion validity with the full dataset. Such psychometric report mainly relies on the COnsensus-based Standards for the selection of health Measurement INstruments (COSMIN) manual and taxonomy (40–42).

#### 2.3.2 Exploratory graph analysis

With the glasso model under Louvain algorithm, bootstrap EGA (bootEGA, iterations = 1,000) was applied to (1) identify a mixing pattern of items, (2) evaluate the stability of items, and (3) discover any possible factor model of the CBCL (36, 43, 44). In EGA plots, each node stands for each item that is attributed to different clusters (i.e., colors); correlating bar plot indicates the stability of each item within factors (unstable if value ≤ 0.650) (43). Both mixing features and stability of items were evaluated as EGA criteria for item removal (**eTable 1 of Supplementary 2**). Structures with two and more factors may be accurately expected over 90% once the sample size has met the threshold (i.e., 500) (43). Regarding structural confirmation, all possible factor models’ output were documented and would be examined in later confirmatory assessment after replication of the structures is stabilized (≥ 0.900 of identified structures) (43). BootEGAs mainly aim to reduce items and explore structures during the instrument forming stage (**eFigure 1 in Supplementary 1**).

#### 2.3.3 Graded response model

Given the ordinal nature of the CBCL, the GRM by Samejima was used to determine three-item aspects, including item discrimination, item characteristic curves, and item information (45–48). GRM parameters served descriptive and evaluative functions for item selection and structural validity assessment (45–48). Essential unidimensionality was evaluated through first/second eigenvalue ratios, the C_2_ statistics, and Yen’s Q3 residual correlations (see eTable notes for detailed indices) (49–51). Discrimination parameters are considered as low, moderate, high, and very high when valued at 0.35–0.64, 0.65–1.34, 1.35–1.69, and higher than 1.70, respectively (45–47). Ideal item characteristic curves should present steeper slopes (discrimination) for each category (e.g., 0 = not true) and clear separation between range from -4.0 to +4.0 on the theta scale (45). In addition, GRM provides item information curves and specific item information values, which complement each other and show items that contribute to the overall informative degree. All three features were set as removal criteria at the instrument forming stage (exploratory assessment; **eTable 1 of Supplementary 2**) and as structural validity indications at the instrument evaluation stage (comprehensive evaluation) to ensure reasonable protocol (**eFigure 1 in Supplementary 1**) (37, 45, 47).

#### 2.3.4 Confirmatory factor analysis

Confirmatory assessments were performed using confirmatory factor analysis (CFA) with a weighted least squares mean- and variance-adjusted (WLSMV) estimation to compare all possible item versions and factor structures for the CBCL (36, 52–54). The item versions include the full 34-item and subsequent reduced versions by bootEGA and GRM. Within these item versions, CFAs were assessed on the one-factor, original five-factor, FFCWS five-factor model, and any new structure output by bootEGA for comparisons of version selection (**eFigure 1 in Supplementary 1**) (13, 14, 25, 28, 52–54). With an adequate sample size using a WLSMV estimator, CFA was reported to contain more accurate estimations (55, 56). Goodness-of-fit (GoF) indices accounting for comparative fit index (CFI), Tucker–Lewis index (TLI), and root mean square error of approximation (RMSEA) were estimated with the following references: CFI ≥ 0.900, TLI ≥ 0.900, and RMSEA ≤ 0.080 (57–63). Models were compared with GoF and its cutoffs to ensure that the model with a relatively better fit was chosen. CFA was conducted in confirmatory and full datasets for structure selection and validity assessments, respectively.

Cross-sectional (CMI) and longitudinal measurement invariance (LMI) were evaluated step by step on the configural, metric, scalar, and strict models (64). The GoFs *per se* and their changes (ΔGoFs for ΔCFI, ΔTLI, and ΔRMSEA) were calculated to determine if measurement invariances are satisfactory (52, 54, 58–61, 65–67). On the one hand, CFI, TLI, and RMSEA were estimated on every model and should be higher than 0.950, higher than 0.950, and lower than 0.010, respectively; on the other hand, |ΔCFI|, |ΔTLI|, and |ΔRMSEA| were compared from the last model (e.g., metric compared with configural model) and favored to be lower than 0.010, lower than 0.010, and lower than 0.015, respectively. CMI and LMI were evaluated in the full dataset.

#### 2.3.5 Internal consistency

Internal consistency was assessed on the revised CBCL and its confirmed structural subscales using the full dataset. Ordinal Cronbach’s alpha (α) and McDonald’s omega (ω), along with their 95% confidence intervals (CIs), were calculated (68–70). A α or ω that is higher than or equal to 0.700 would be considered satisfactory as a reliability index (66, 71).

#### 2.3.6 Receiver operating characteristics

The receiver operating characteristic (ROC) analysis approach was deployed to determine the criterion validity of the revised CBCL (72, 73). Versus an ADHD binary diagnosis by clinical professionals in full data, ROC curves were plotted on CBCL total scores and sub-scores by the true positive rate (sensitivity; sensitivity = true positive count/[true positive count + false negative count]) and false positive rate (1 - specificity; specificity = true negative count/[true negative count + false positive count]) (74). Area under the curve (AUC) was subsequently computed to signify that diagnostic efficiency is acceptable (0.7 ≤ value < 0.8), excellent (0.8 ≤ value < 0.9), and outstanding (value ≥ 0.9) (73).

## 3 Results

### 3.1 Participants

A total of 1,786 records were included, with a balanced sex ratio of 49.5% (*n* = 884) female. The majority of youths (68.6%, *n* = 1,226) were 15 years or younger at T3. In terms of races, 51.4% (*n* = 918) of the youth identified themselves as Black/African American, 19.2% (*n* = 343) as White, and 29.4% (*n* = 525) as other racial backgrounds. The youth who met the clinical diagnostic criteria for ADHD was 16.3% (*n* = 291). Other detailed information are summarized in Supplement 3.

### 3.2 Item reduction and model selection

During the initial round of item removal, items 8, 9, 10, 11, 14, 18, 30, 33, and 34 exhibited low item stability more than twice (<0.651), which met the EGA removal criteria (**eFigure 2 of Supplementary 1**). Subsequent GRM results indicated that items 1, 4, 8, 17, 18, 24, 26, 27, and 28 have fulfilled more than two removal criteria regarding discrimination (**eTable 2 of Supplementary 2**), characteristics (**eFigure 3–5 of Supplementary 1**), and information (**eFigures 6–8 of Supplementary 1**; **eTable 2 of Supplementary 2**). Furthermore, we re-evaluated these items that fell into several conceptual considerations for removal: 1) anxiety and depressive items (i.e., “clings to adults”, “worries”, “cries a lot”) representing a distinct psychopathological dimension with low discrimination for assessment; 2) low base-rate conduct behaviors (i.e., “runs away from home”, “sets fires”) exhibiting narrow properties due to extreme rarity; 3) non-specific hyperactivity indicators (i.e., “talks too much”, “unusually loud”) showing unstable factor attribution across timepoints; 4) affective reactivity items (i.e., “argues a lot”, “stubborn/irritable”, “temper tantrums”) reflecting normative variability rather than stable behavioral indicators; and 5) covert antisocial behaviors (i.e., “steals at home”, “swears/obscene language”) exhibiting developmental instability. Full item contents are provided in **eTable 2 of Supplementary 2**. Taking the undesirable properties of the items listed above into consideration, a total of 17 items (pool nos. 1, 4, 8, 9, 10, 11, 12, 14, 17, 18, 24, 26, 27, 28, 30, 33, and 34) were selected for initial removal (**eTable 3 of Supplementary 2**).

The remaining 17 items were included and recoded for the subsequent second round of reduction analyses. Illustrated by **eFigure 9 of Supplementary 1**, items 9 (initially no. 19), 10 (initially no. 20), 14 (initially no. 25), and 15 (initially no. 25) did not contain adequate stability features and therefore met the removal standard. Furthermore, items 1 (initially no. 2), 2 (initially no. 3), 11 (initially no. 21), and 14 (initially no. 25) have represented unsatisfactory GRM properties more than twice covering discrimination (**eTable 4 of Supplementary 2**), characteristics (**eFigures 10–12 of Supplementary 1**), and information (**eFigures 13–15 of Supplementary 1**; **eTable 4 of Supplementary 2**). With the second round of dual item assessments, a total of seven items (initial pool nos. 2, 3, 19, 20, 21, 25, and 29) listed above should be removed (**eTable 5 of Supplementary 2**). The second-round-eliminated items also primarily represented anxiety/depressive (e.g., “nervous/highstrung/tense”) and covert antisocial behaviors (e.g., “steals at home”) items (**eTable 4 of Supplementary 2**).

After removing 24 items from the initial pool, a 10-item CBCL pool was generated for the third removal evaluation. Compared with previous fitting solution percentages of bootstrap iterations (34-item = 50.3%–56.0%; 17-item = 48.1%–65.3%), the 10-item version revealed satisfactory cluster replication with values of 92.2% at T1, 99.4% at T2, and 99.8% at T3. The items were mostly stable with values ranging from 0.710 to 1.000 (**eFigure 16 of Supplementary 1**). Considering GRM indices, the 10-item CBCL pool displayed moderate to very high discrimination [*a* = 1.238–2.754 (T1), 1.807–2.863 (T2), and 1.766–3.713 (T3); **eTable 6 of Supplementary 2**], which were in accordance with item characteristic curves (**eFigures 17–19 of Supplementary 1**). All 10 items, as well as the information curves depicted, contained sufficient information valued from 1.971 to 6.939 (**eFigure 20–22 of Supplementary 1**; **eTable 6 of Supplementary 2**).

Given all the satisfactory properties demonstrated by the 10-item pool, the reduction procedures were terminated and confirmatory assessments were initiated (**eTables 7, 8 of Supplementary 2**). The 10-item version of the CBCL has delineated more robust fit than the 34-item version, as indicated by CFIs (0.857–0.990 vs. 0.599–0.963), TLIs (0.816–0.986 vs. 0.572–0.960), and RMSEAs (0.043–0.105 vs. 0.030–0.107). Within the 10-item version, two EGA three-factor structures (CFI = 0.931–0.990, TLI = 0.903–0.986, RMSEA = 0.043–0.076) were fitted relatively better than three other structures (CFI = 0.857–0.989, TLI = 0.816–0.985, RMSEA = 0.046–0.105). Notably, the only distinction between the two EGA three-factor structures was the attribution of item 3 (initial pool no. 7; “Youth is impulsive or acts without thinking”). This item was attributed to AP at T2 and T3 but to OD at T1. We selected the EGA structure from T2 and T3, given that impulsivity represents a core feature of ADHD rather than oppositional defiant behavior.

In summary, the CBCL was reduced from 34 items to 10 items (CBCL-10) with a three-factor model. We named the three factors (subscales) as *attention problems (AP), threatening behavior (TB)*, and *oppositional defiant (OD)* based on original naming and item content. The retained 10 items map coherently onto established theoretical frameworks. The *AP* subscale (three items: “can’t concentrate”, “can’t sit still/hyperactive”, “impulsive”) directly assesses ADHD core symptoms accounting for inattention, hyperactivity, and impulsivity symptoms. The *TB* subscale (four items: “gets in many fights”, “physically attacks”, “threatens people”, “vandalizes”) captures aggressive and destructive behaviors characteristic of conduct problems frequently comorbid with ADHD. The *OD* subscale (three items: “lies or cheats”, “disobedient at home”, “disobedient at school”) assesses rule violation and noncompliance across settings. The CBCL-10 was subsequently evaluated on overall psychometric properties using full dataset.

### 3.3 Overall properties

#### 3.3.1 Structural validity

**Figure 1:**
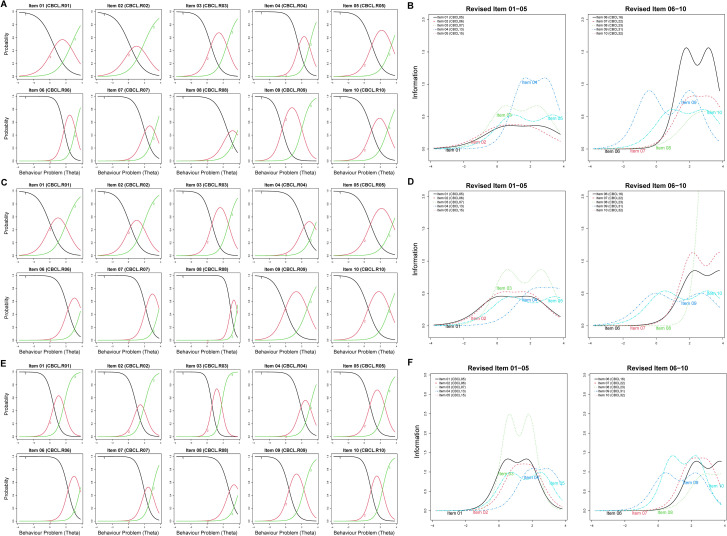
Visualization of the final 10-item CBCL GRM parameters (full data, *N* = 1,786): **(A)** item characteristic curves (T1), **(B)** item information curves (T1), **(C)** item characteristic curves (T2), **(D)** item information curves (T2), **(E)** item characteristic curves (T3), and **(F)** item information curves (T3). Differently colored curves represent different options/responses for the item. CBCL, Child Behavior Checklist; CBCL.R, revised Child Behavior Checklist.

**Figure 2:**
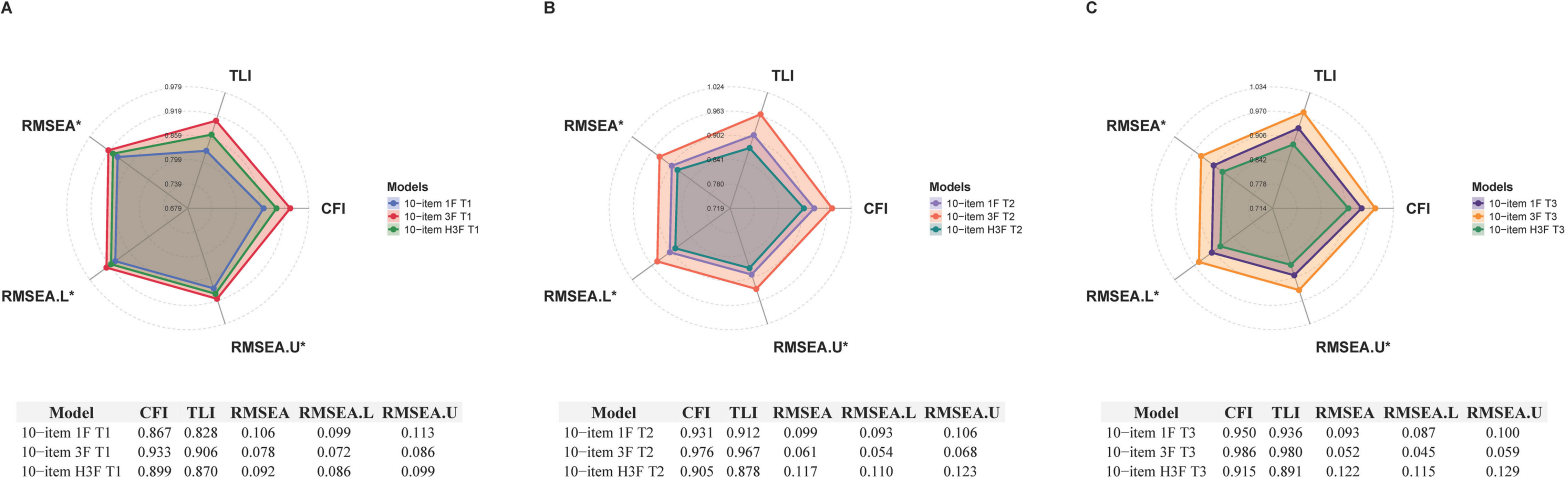
Radar charts of GoF comparisons on factorial models at **(A)** T1, **(B)** T2, and **(C)** T3. RMSEA and its confidence intervals have been reversed (1−RMSEA) for better presentation in radar charts. Higher values of CFI and TLI and lower values of RMSEA and its confidence intervals indicate better model fit. CFI, comparative fit index; TLI, Tucker−Lewis index; RMSEA, root mean square error of approximation; RMSEA.L, lower interval of the RMSEA; RMSEA.U, upper interval of the RMSEA; 1F, one-factor; 3F, three-factor; H3F, higher order three-factor; T1, time-point 1; T2, time-point 2; T3, time-point.

The CBCL-10 possesses ideal characteristic curves and moderate to very high item discrimination in all three time points (*a* = 1.144–2.472 [T1], 1.756–2.914 [T2], and 1.899–3.139 [T3]; **Figures 1A, C, E**). No item of CBCL-10 showed low or relatively low information values or curve levels (**Figures 1B, D, F**). Furthermore, GoF plots portrayed that the three-factor model fitted relatively better than one-factor and higher-order three-factor models (**Figure 2**). The CBCL-10 illustrated adequate structural validity regarding aspects of GRM (**eTable 9 of Supplementary 2**) and GoF indices.

#### 3.3.2 Measurement invariances

The GoF indices of CMI, stratified by the subgroups of youth sex, youth age, and youth ADHD diagnosis status, were all satisfactory since the values have met the expectations (CFI = 0.932–0.990, TLI = 0.905–0.990, RMSEA = 0.044–0.078; **Table 1**). Besides that, most ΔGoFs did not fall out of the cutoffs when constraints were added to invariance models (**Table 1**). Moreover, the LMI have shown adequate fit indices with CFI ranging from 0.964 to 0.976, TLI ranging from 0.958 to 0.971, and RMSEA ranging from 0.030 to 0.037. The majority of the changes within LMI indexes were acceptable or near acceptable, which further supported the invariances across time points (**Table 1**). These results suggest that CBCL-10 can be considered invariant when tested across youth sex, age, ADHD diagnosis status, and time points.

**Table 1:** Fit indices of cross-sectional and longitudinal measurement invariance of the CBCL across subgroups and time points (full data, *N* = 1,786).

Model	*χ*^2^ (*df*)	*P*	Scaled chi-square difference statistics		CFI	ΔCFI	TLI	ΔTLI	RMSEA (90% CI)	ΔRMSEA
			Δ*χ*^2^ (Δ*df*)	*P*						
Sex at T1										
Configural	411.881 (64.)	<0.001	/	/	0.932	/	0.905	/	0.078 (0.071, 0.085)	/
Metric	403.691 (71.)	<0.001	9.488 (7)	0.220	0.935	0.003	0.918	0.013	0.073 (0.066, 0.079)	-0.006
Scalar	387.063 (78.)	<0.001	11.86 (7)	0.105	0.940	0.005	0.931	0.013	0.067 (0.060, 0.073)	-0.006
Strict	373.876 (88.)	<0.001	5.159 (10)	0.880	0.944	0.005	0.943	0.013	0.060 (0.054, 0.067)	-0.006
Sex at T2										
Configural	274.778 (64.)	<0.001	/	/	0.976	/	0.966	/	0.061 (0.053, 0.068)	/
Metric	284.122 (71.)	<0.001	11.442 (7)	0.120	0.976	0.000	0.969	0.003	0.058 (0.051, 0.065)	-0.003
Scalar	277.180 (78.)	<0.001	12.496 (7)	0.085	0.977	0.002	0.974	0.005	0.054 (0.047, 0.060)	-0.005
Strict	277.106 (88.)	<0.001	18.484 (10)	0.047	0.978	0.001	0.978	0.004	0.049 (0.043, 0.056)	-0.004
Sex at T3										
Configural	205.574 (64.)	<0.001	/	/	0.987	/	0.982	/	0.050 (0.042, 0.058)	/
Metric	211.822 (71.)	<0.001	10.683 (7)	0.153	0.987	0.000	0.983	0.002	0.047 (0.040, 0.055)	-0.003
Scalar	220.413 (78.)	<0.001	21.249 (7)	0.003	0.987	0.000	0.985	0.001	0.045 (0.038, 0.052)	-0.002
Strict	238.031 (88.)	<0.001	26.735 (10)	0.003	0.986	-0.001	0.986	0.001	0.044 (0.037, 0.050)	-0.002
Youth latest age at T3										
Configural	218.407 (64.)	<0.001	/	/	0.986	/	0.980	/	0.052 (0.045, 0.060)	/
Metric	212.197 (71.)	<0.001	4.644 (7)	0.703	0.987	0.001	0.983	0.004	0.047 (0.040, 0.055)	-0.005
Scalar	198.445 (77.)	<0.001	6.026 (6)	0.420	0.989	0.002	0.987	0.003	0.042 (0.035, 0.049)	-0.005
Strict	192.048 (87.)	<0.001	8.291 (10)	0.600	0.990	0.002	0.990	0.003	0.037 (0.030, 0.044)	-0.005
Youth race at T3										
Configural	226.459 (96.)	<0.001	/	/	0.988	/	0.984	/	0.048 (0.040, 0.056)	/
Metric	218.638 (110)	<0.001	10.723 (14)	0.708	0.990	0.002	0.988	0.004	0.041 (0.033, 0.049)	-0.007
Scalar	253.711 (120)	<0.001	38.491 (10)	< 0.001	0.988	-0.002	0.987	-0.002	0.043 (0.036, 0.051)	0.003
Strict	269.505 (140)	<0.001	24.016 (20)	0.242	0.988	0.000	0.989	0.002	0.039 (0.032, 0.047)	-0.004
Youth ADHD diagnosis at T3										
Configural	192.927 (64.)	<0.001	/	/	0.979	/	0.971	/	0.048 (0.040, 0.055)	/
Metric	209.390 (71.)	<0.001	20.105 (7)	0.005	0.978	-0.002	0.972	0.001	0.047 (0.039, 0.054)	-0.001
Scalar	241.103 (77.)	<0.001	30.582 (6)	< 0.001	0.974	-0.004	0.969	-0.003	0.049 (0.042, 0.056)	0.002
Strict	250.862 (87.)	<0.001	22.452 (10)	0.013	0.974	0.000	0.973	0.004	0.046 (0.039, 0.053)	-0.003
Youth depression diagnosis at T3										
Configural	202.869 (64.)	<0.001	/	/	0.985	/	0.978	/	0.049 (0.042, 0.057)	/
Metric	215.866 (71.)	<0.001	18.274 (7)	0.011	0.984	-0.001	0.980	0.001	0.048 (0.041, 0.055)	-0.001
Scalar	208.185 (77.)	<0.001	6.442 (6)	0.376	0.985	0.002	0.983	0.003	0.044 (0.037, 0.051)	-0.004
Strict	216.125 (87.)	<0.001	18.016 (10)	0.055	0.986	0.000	0.985	0.002	0.041 (0.034, 0.048)	-0.003
Longitudinal (T1, T2, and T3)										
Configural	933.405 (339)	<0.001	/	/	0.976	/	0.969	/	0.031 (0.029, 0.034)	/
Metric	937.990 (353)	<0.001	11.319 (14)	0.661	0.976	0.000	0.971	0.002	0.030 (0.028, 0.033)	-0.001
Scalar^a^	1,240.497 (367)	<0.001	246.625 (14)	< 0.001	0.965	-0.012	0.958	-0.013	0.037 (0.034, 0.039)	0.006
Strict	1,268.112 (387)	<0.001	90.414 (20)	< 0.001	0.964	0.000	0.960	0.002	0.036 (0.034, 0.038)	-0.001

*χ*^2^, chi-square; *df*, degrees of freedom; CFI, comparative fit index; TLI, Tucker–Lewis index; RMSEA, root mean square error of approximation; CI, confidence interval; Δ, a change in *χ*^2^, df, CFI, TLI, and RMSEA.

^a^This model is nearly supported.

#### 3.3.3 Internal consistency

Based on the three-factor model, the internal consistency of CBCL-10 was decent with α value of 0.880–0.934, while other subscales including *AP, TB,* and *OD* had values of 0.732–0.907, 0.816–0.911, and 0.776–0.838, respectively (**eTable 10 of Supplementary 2**). The ω value further supported the evidence of robust internal consistency, with CBCL-10 ranging 0.880–0.933, AP ranging 0.754–0.908, TB ranging 0.823–0.915, and *OD* ranging 0.781–0.855 (**eTable 10 of Supplementary 2**).

#### 3.3.4 Criterion validity

**Figure 3:**
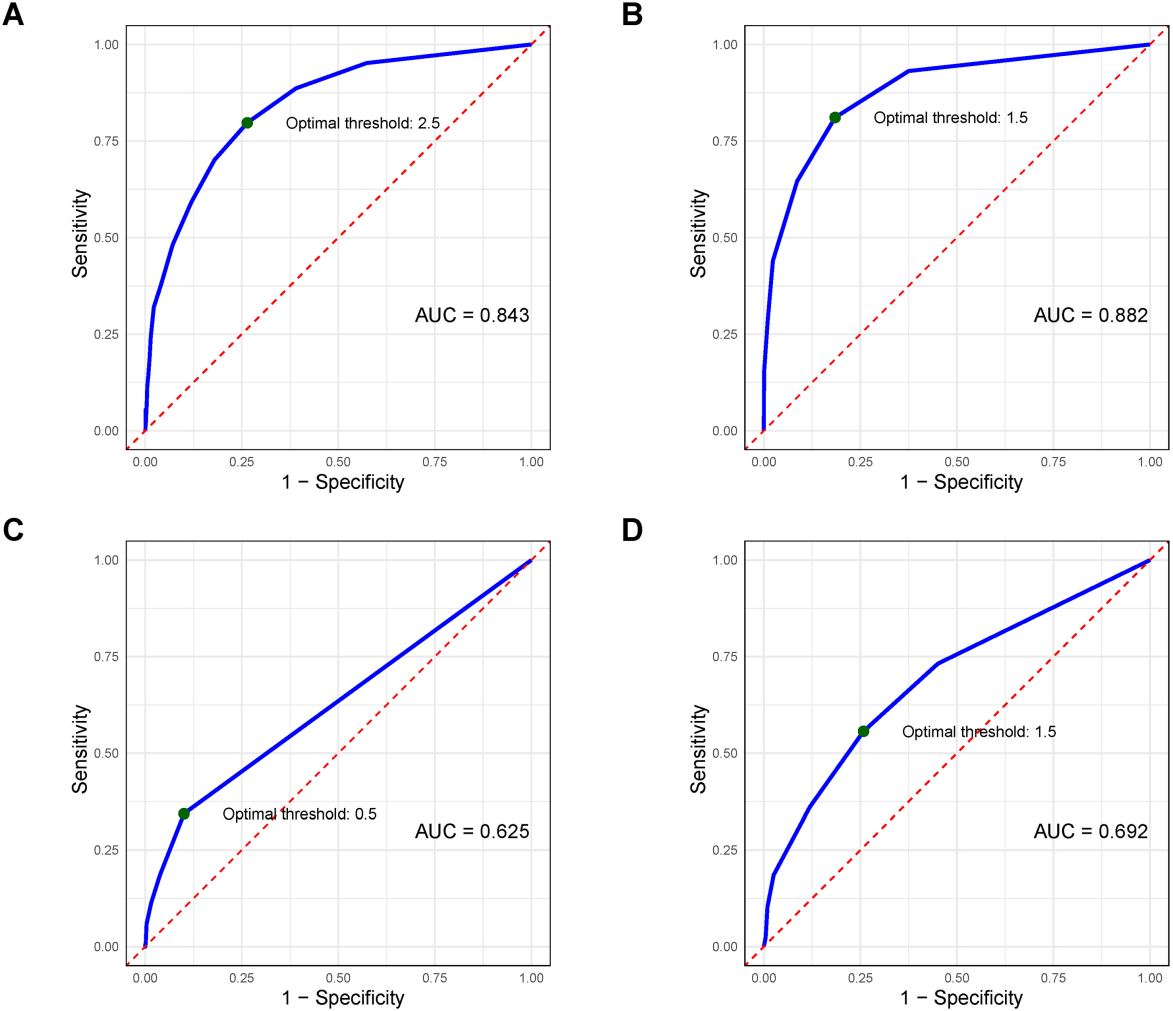
ROC curves of **(A)** CBCL, **(B)** AP, **(C)** TB, and **(D)** OD scores on ADHD (full data, *N* = 1,786). The green point indicates the optimal cutoff value of measurement. The diagonal red line represents the line of no discrimination. AUC, area under the curve; ROC curve, receiver operating characteristic curve; CBCL, Child Behavior Checklist; AP, attention problems; TB, threatening behavior; OD, oppositional defiant.

The overall criterion validity, determined by ROC analyses (**Figure 3A**), was diagnostically efficient given that the AUC of CBCL-10 was 0.843 (95% CI = 0.818–0.867). CBCL-10 had an optimal diagnostic score of 2.5 (i.e., 3), with the optimal specificity and sensitivity values being 0.736 and 0.797, respectively. As for the subscales of *AP* (**Figure 3B**), *TB* (**Figure 3C**), and *OD* (**Figure 3D**), the diagnostic efficiency was excellent (AUC [95% CI] = 0.882 [0.860–0.904]), unacceptable (AUC [95% CI] = 0.625 [0.597–0.654]), and unacceptable (AUC [95% CI] = 0.692 [0.658–0.726]), respectively. The ideal *AP* diagnostic score was 1.5 (i.e., 2) if applied as a screening tool. Generally, CBCL-10 contains sufficient criterion validity.

## 4 Discussion

### 4.1 Item and model selection

Through a standardized multi-stage process, we successfully reduced the original 34-item CBCL to a concise 10-item version while maintaining strong psychometric properties. This substantial reduction in scale length addresses the practical limitations associated with ADHD screening tools in clinical and research settings (20, 22, 24, 75). The trade-off between brevity and comprehensiveness is an inherent consideration in scale reduction efforts (76, 77). While CBCL-10 demonstrates strong psychometric properties, clinicians and researchers should remain aware that the abbreviated scale may not capture the full spectrum of attention and behavioral problems assessed by the original CBCL (13, 18).

The decision to place item 3 (“Youth is impulsive or acts without thinking”) into the *AP* factor rather than the *OD* factor was theoretically justified—impulsivity is a core feature of attention deficit symptoms (14, 78, 79). This careful consideration of item content during the reduction process helped ensure that the resulting scale maintained conceptual integrity while optimizing statistical properties (38, 39, 80).

The final 10-item measurement demonstrated an excellent model fit that was more improved than the full 34-item version, as evidenced by superior GoF indices (25, 28). Notably, the three-factor structure that emerged from the current study provides a theoretically sound framework for understanding youth attention and detailed behavioral problems, categorizing items into *AP, TB,* and *OD* domains (1, 13–15, 22). This structural model aligns with previous conceptualizations of attention and externalizing problems in youth, particularly those relevant to ADHD and related behavioral disorders (2, 4, 14, 78).

### 4.2 Measurement invariances

A critical strength of CBCL-10 is its demonstrated measurement invariance across key demographic and clinical characteristics. These findings indicate that CBCL-10 measures the same constructs in the same way across youth sex, youth age, race/ethnicity, and ADHD diagnostic status, which is essential for valid group comparisons in clinical research and practice (81, 82). Specifically, the successful establishment of measurement invariance across ADHD diagnostic groups confirms that the three-factor structure functions equivalently in both clinical and non-clinical groups, supporting the interpretability of ROC-based criterion validity analyses. The LMI of CBCL-10 further underscores its utility for tracking attention/behavioral changes over time, which is valuable for longitudinal studies and clinical monitoring (25, 36, 38, 75). It ensures that observed changes in scores reflect true trends rather than measurement bias.

### 4.3 Diagnose property

The diagnostic efficiency evidenced by ROC analyses indicated the good screening ability of CBCL-10 between youth with and without ADHD diagnoses (83, 84). An optimal cutoff score of 3 provides balanced sensitivity and specificity, which supports the tool’s utility as an efficient screening measure. Notably, the AP subscale exhibited excellent diagnostic performance with an optimal screening cutoff score of 2. This superior performance of the AP subscale is consistent with its theoretical alignment with ADHD symptomatology and suggests that it could potentially serve as a standalone screening tool for attention problems (1, 13, 14). In contrast, the *TB* and *OD* subscales showed unacceptable diagnostic efficiency for ADHD. This may be attributed to the fact that these domains capture behavioral problems that are often comorbid with ADHD, which are not core diagnostic features of the disorder (1, 3, 14, 78, 79). The identified cutoff scores of CBCL-10 provide clinically meaningful thresholds that can guide referral decisions and treatment planning in various settings.

Moreover, the false negative cases (youth with ADHD diagnoses who scored below the cutoff) exhibited a distinctive symptom profile: these individuals suggest that CBCL-10 may be less sensitive to predominantly inattentive presentations that lack behavioral manifestations. This finding aligns with the known variety of ADHD presentations and suggests that clinicians should consider detailed assessment for youth presenting with attention difficulties in the absence of disruptive behaviors. Unfortunately, the FFCWS dataset does not include ADHD subtype information, precluding direct examination of whether false negatives disproportionately represent the predominantly inattentive presentation. Future validation studies can examine the performance of CBCL-10 across ADHD subtypes to inform clinical decision-making.

### 4.4 Strengths and limitations

This study has several noteworthy advantages. First, our large and diverse sample with balanced sex representation and substantial racial diversity provided enhanced generalizability of our findings (25, 28). Second, the application of contemporary approaches, including EGA and GRM, allowed for standardized item reduction that optimized both theoretical and statistical considerations (37, 45–48). Third, this reduction in length (from 34 to 10 items) is achieved without substantially sacrificing psychometric integrity, which represented a meaningful advancement in efficient clinical screening (77, 80). Fourth, the comprehensive evaluation of measurement invariance across multiple dimensions provides robust evidence for the broad applicability of the measurement.

Despite strengths, several limitations still exist. First, while our sample was demographically diverse, it may not fully represent all clinical populations or cultural contexts, potentially limiting extrapolatability. Although we employed a two-stage process, external validation in independent samples and diverse clinical populations is warranted to establish generalizability. Second, the study relied exclusively on parent-reported behavior, without incorporating teacher reports or self-reports, which could provide complementary perspectives (85). Third, the substantial item reduction, particularly not starting from the complete 113-item version, inevitably results in some loss of content coverage compared to the original scale, potentially reducing the measure’s ability to capture nuanced attention problems, mental symptoms, and behavioral presentations (14). Lastly, the ADHD diagnosis was determined at a single time point, with some individuals showing symptom remission while others exhibit persistent or late-emerging presentations (2). This means that some youth classified as ADHD at T3 may not have met the criteria at earlier time points and vice versa. Future studies with repeated diagnostic assessments across waves would enable a more nuanced examination of diagnostic stability.

### 4.5 Future directions

Several routes for future research emerge from this study. First, external validation of CBCL-10 in other independent samples and diverse clinical settings would strengthen the evidence for its generalizability. Second, investigation of the scale’s responsiveness to intervention would establish its utility as an outcome measure in ADHD treatment studies. Third, examination of CBCL-10’s relationship with biomarkers, neuropsychological performance, and functional outcomes would enhance the understanding of its construct validity and clinical significance. Finally, standardized item reduction based on the full 113-item original CBCL is worth trying to ensure that other critical features (e.g., mental symptoms) can be retained.

## 5 Conclusions

The present study successfully developed a psychometrically sound 10-item version of CBCL through a standardized item reduction approach. The reduced length of CBCL-10 addresses practical limitations in clinical settings. With established cutoff scores for clinical screening, CBCL-10 represents a valuable addition to the toolkit of clinicians and researchers for measuring youth attention/behavioral problems without sacrificing measurement quality.

## Data Availability

The datasets presented in this study can be found in online repositories. Researchers can apply to access the data used in this study from the Future of Families and Child Wellbeing Study website (https://ffcws.princeton.edu/).
