# Supplementary material for: Measurement properties of the Child Behavior Checklist-10: an ultra-brief screening updated in longitudinal cohort
Source: Front Psychiatry. 2026 Apr 30;17:1767665. doi: 10.3389/fpsyt.2026.1767665 (PMC13171534; doi:10.3389/fpsyt.2026.1767665)
Supplement: Supplementary file 1 [file DataSheet1.pdf]

Supplement 1_eFigures

Index

eFigure 1. Flow chart of the study protocol.

eFigure 2. Item clusters and stability of the 34-item pool at A) T1, B) T2 and, C) T3 (Exploratory set, *N* = 893).

eFigure 3. Item characteristics of the 34-item pool at T1 (Exploratory set, *N* = 893).

eFigure 4. Item characteristics of the 34-item pool at T2 (Exploratory set, *N* = 893).

eFigure 5. Item characteristics of the 34-item pool at T3 (Exploratory set, *N* = 893).

eFigure 6. Item information curves of the 34-item pool at T1 (Exploratory set, *N* = 893).

eFigure 7. Item information curves of the 34-item pool at T2 (Exploratory set, *N* = 893).

eFigure 8. Item information curves of the 34-item pool at T3 (Exploratory set, *N* = 893).

eFigure 9. Item clusters and stability of the 17-item pool at A) T1, B) T2 and, C) T3 (Exploratory set, *N* = 893).

eFigure 10. Item characteristics of the 17-item pool at T1 (Exploratory set, *N* = 893).

eFigure 11. Item characteristics of the 17-item pool at T2 (Exploratory set, *N* = 893).

eFigure 12. Item characteristics of the 17-item pool at T3 (Exploratory set, *N* = 893).

eFigure 13. Item information curves of the 17-item pool at T1 (Exploratory set, *N* = 893).

eFigure 14. Item information curves of the 17-item pool at T2 (Exploratory set, *N* = 893).

eFigure 15. Item information curves of the 17-item pool at T3 (Exploratory set, *N* = 893).

eFigure 16. Visualized structural network of the second round revised CBCL (Exploratory data, N = 893).

eFigure 17. Item characteristics of the 10-item pool at T1 (Exploratory set, *N* = 893).

eFigure 18. Item characteristics of the 10-item pool at T2 (Exploratory set, *N* = 893).

eFigure 19. Item characteristics of the 10-item pool at T3 (Exploratory set, *N* = 893).

eFigure 20. Item information curves of the 10-item pool at T1 (Exploratory set, *N* = 893).

eFigure 21. Item information curves of the 10-item pool at T2 (Exploratory set, *N* = 893).

eFigure 22. Item information curves of the 10-item pool at T3 (Exploratory set, *N* = 893).

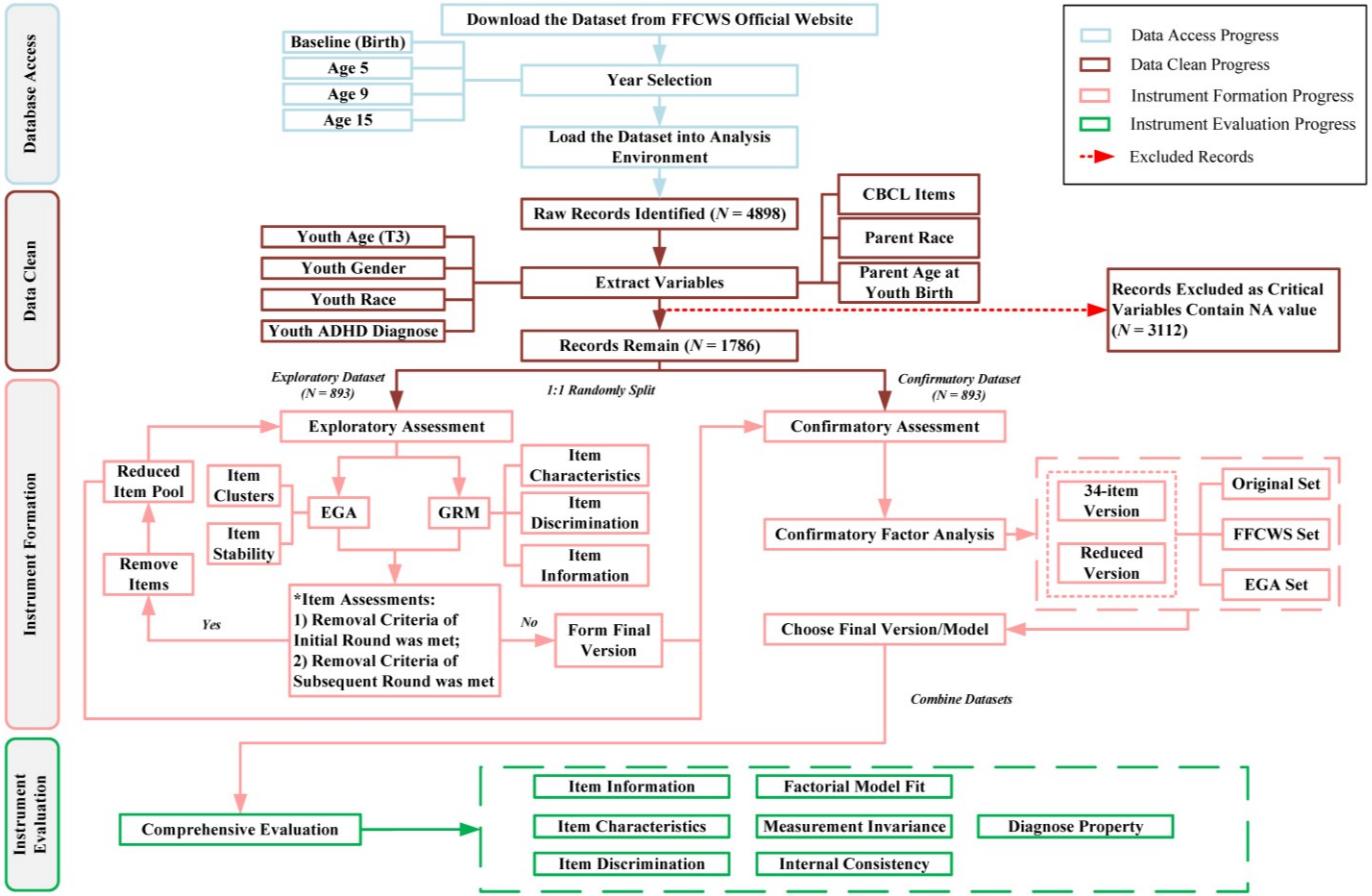

eFigure 1. Flow chart of the study protocol.

*Removal Criteria was described in Supplement 2.

Abbreviations: *FFCWS* The Future of Families and Child Wellbeing Study, *CBCL* Child Behavior Checklist, *EGA* Exploratory Graph Analysis, *GRM* Graded Response Model.

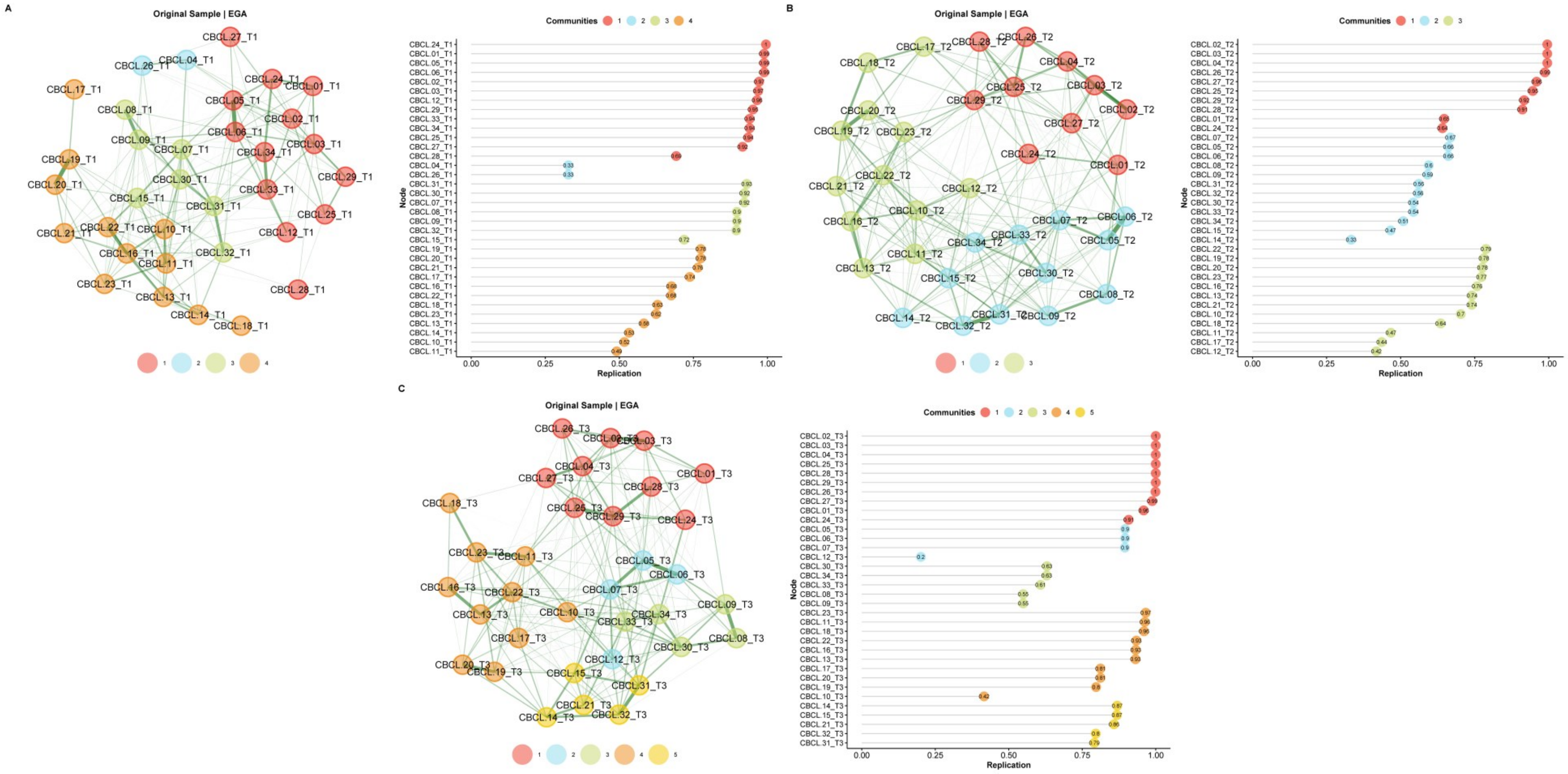

eFigure 2. Item clusters and stability of the 34-item pool at A) T1, B) T2 and, C) T3 (Exploratory set, $N = 893$).

The overall output stabilities of the 34-item pool structures were valued at only 56.0%, 50.3%, and 52.2% at T1, T2, and T3, respectively.

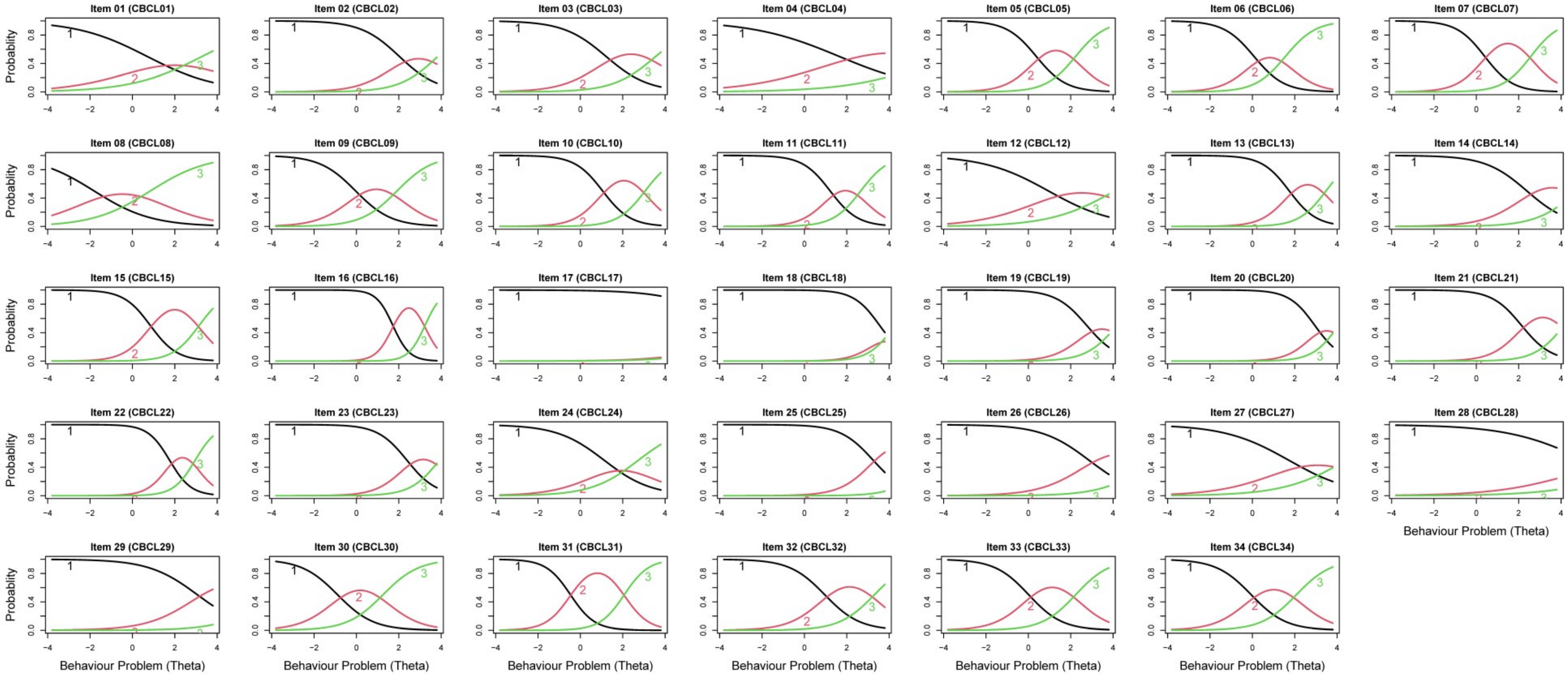

eFigure 3. Item characteristics of the 34-item pool at T1 (Exploratory set, $N = 893$).

Abbreviations: *CBCL* Child Behavior Checklist.

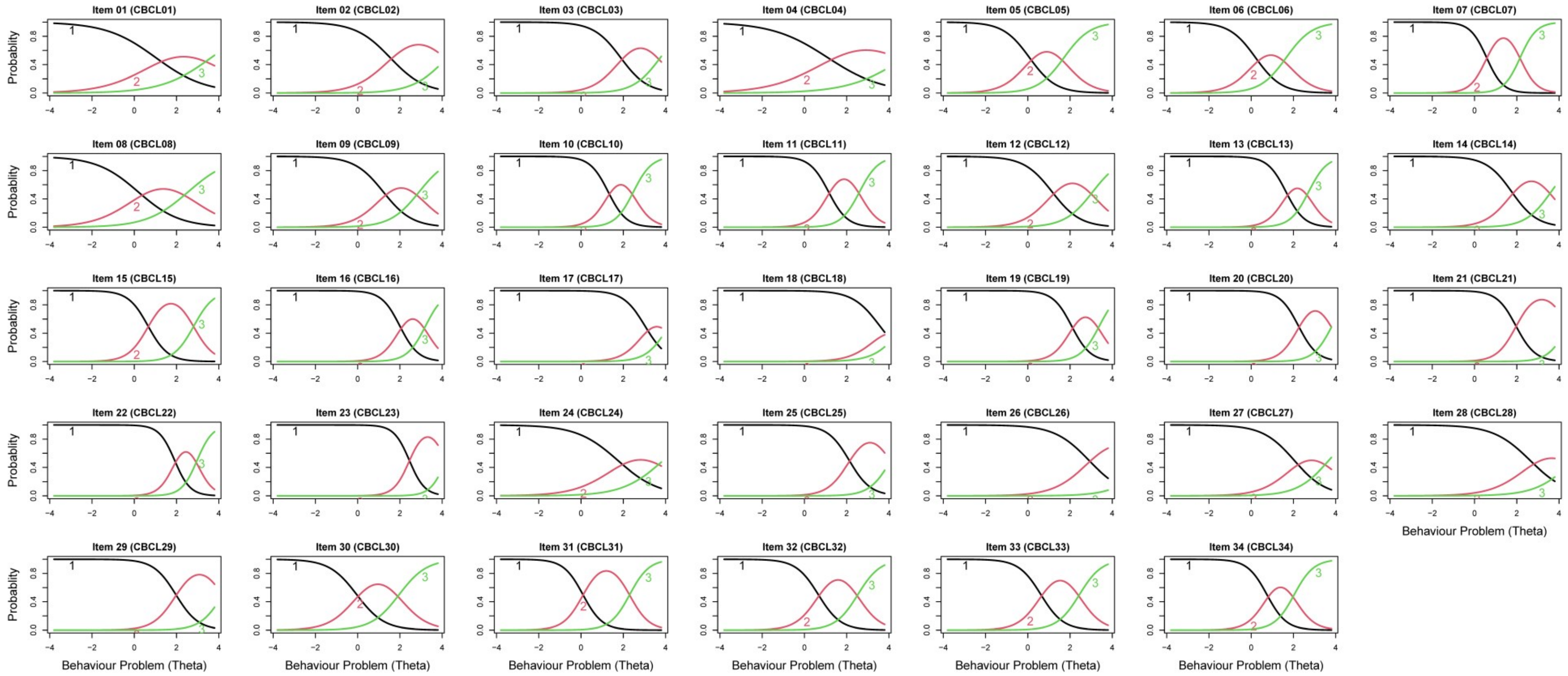

eFigure 4. Item characteristics of the 34-item pool at T2 (Exploratory set, *N* = 893).

Abbreviations: *CBCL* Child Behavior Checklist.

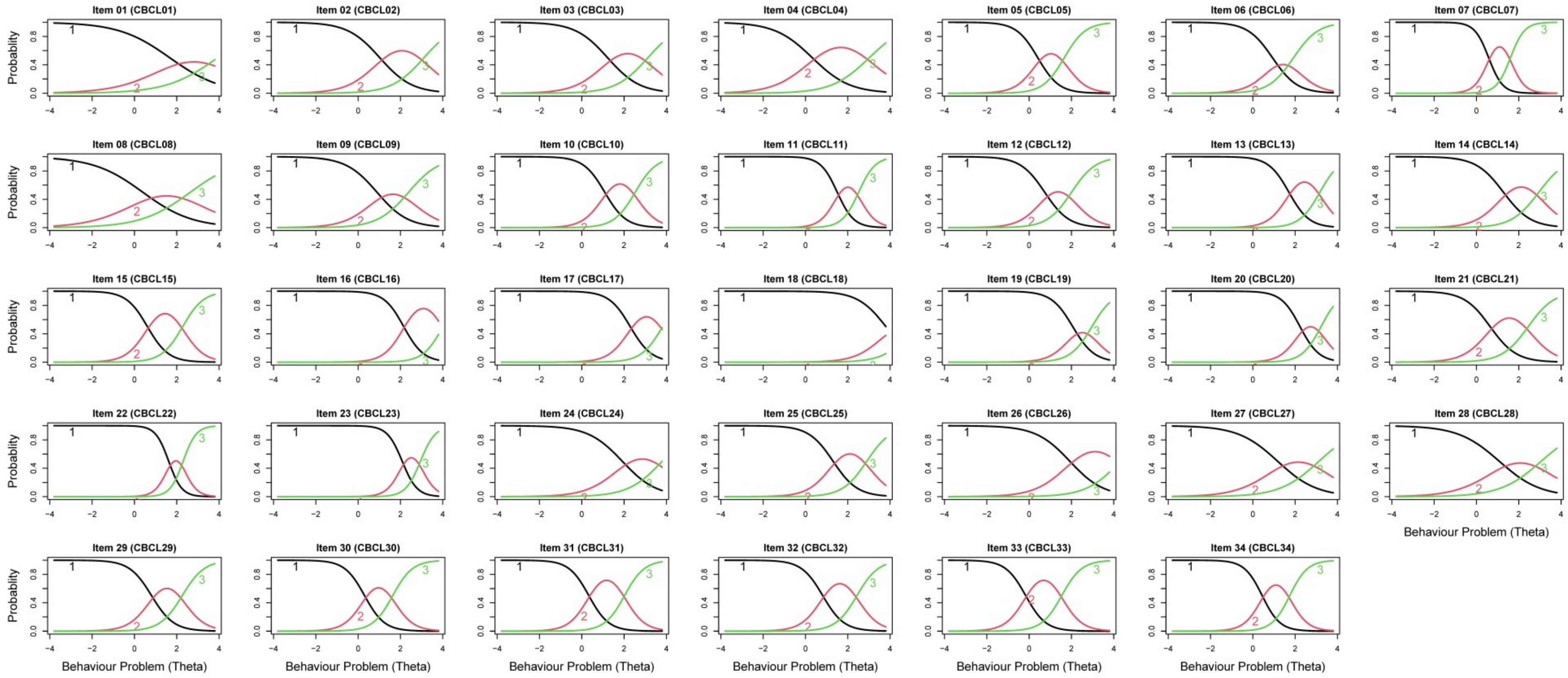

eFigure 5. Item characteristics of the 34-item pool at T3 (Exploratory set, N = 893).

Abbreviations: *CBCL* Child Behavior Checklist.

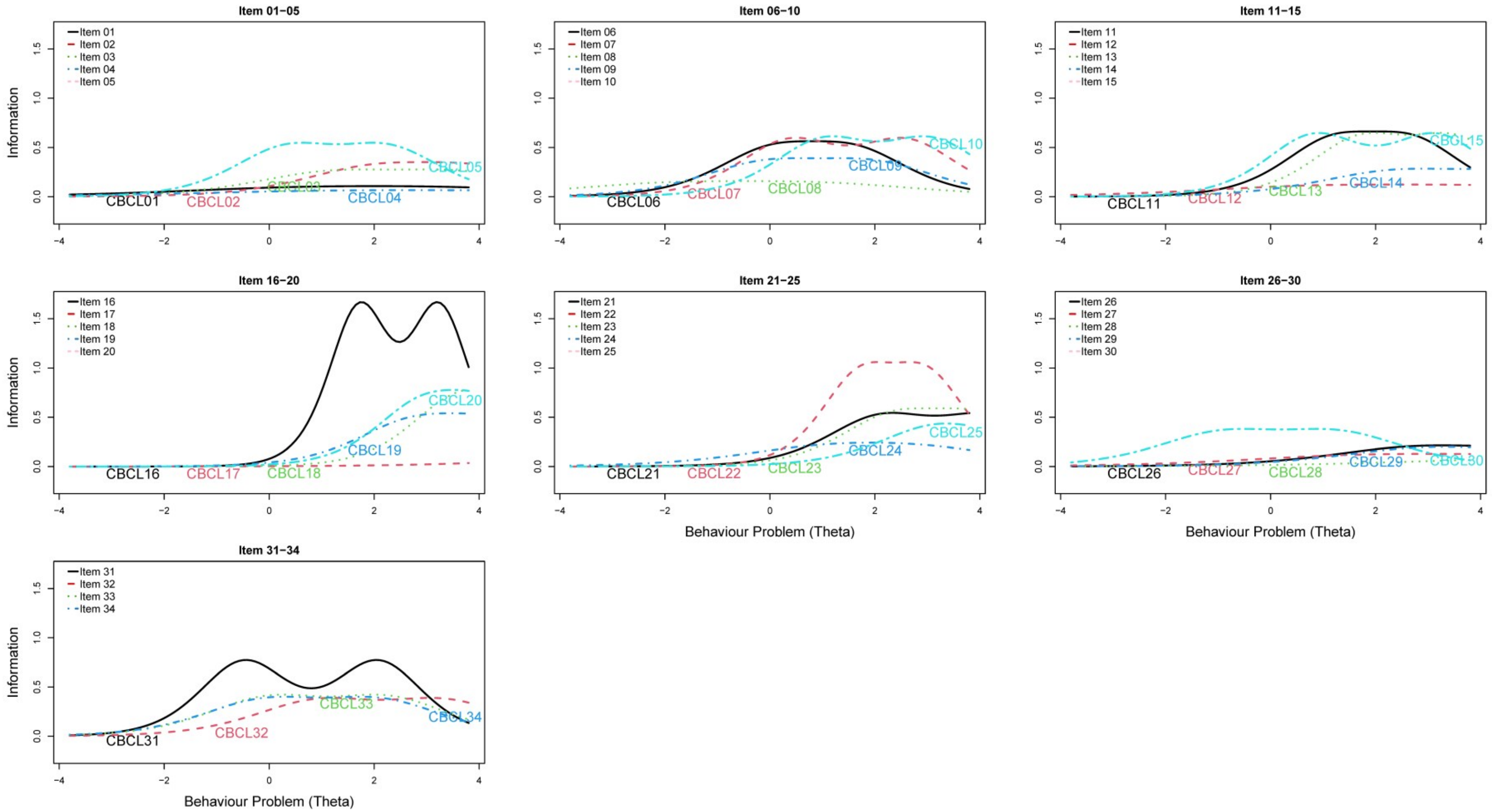

eFigure 6. Item information curves of the 34-item pool at T1 (Exploratory set, $N = 893$).

Abbreviations: *CBCL* Child Behavior Checklist.

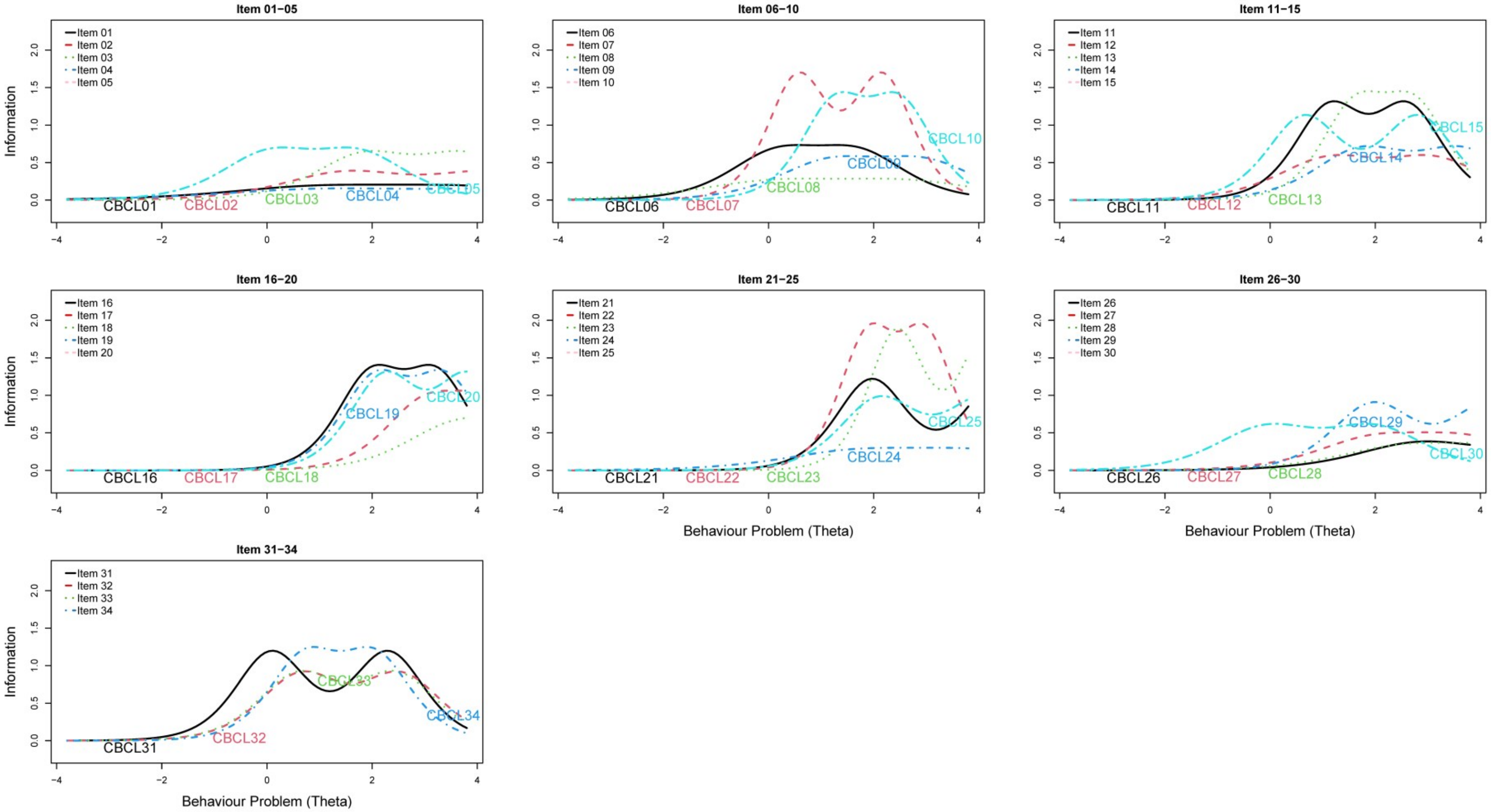

eFigure 7. Item information curves of the 34-item pool at T2 (Exploratory set, $N = 893$).

Abbreviations: *CBCL* Child Behavior Checklist.

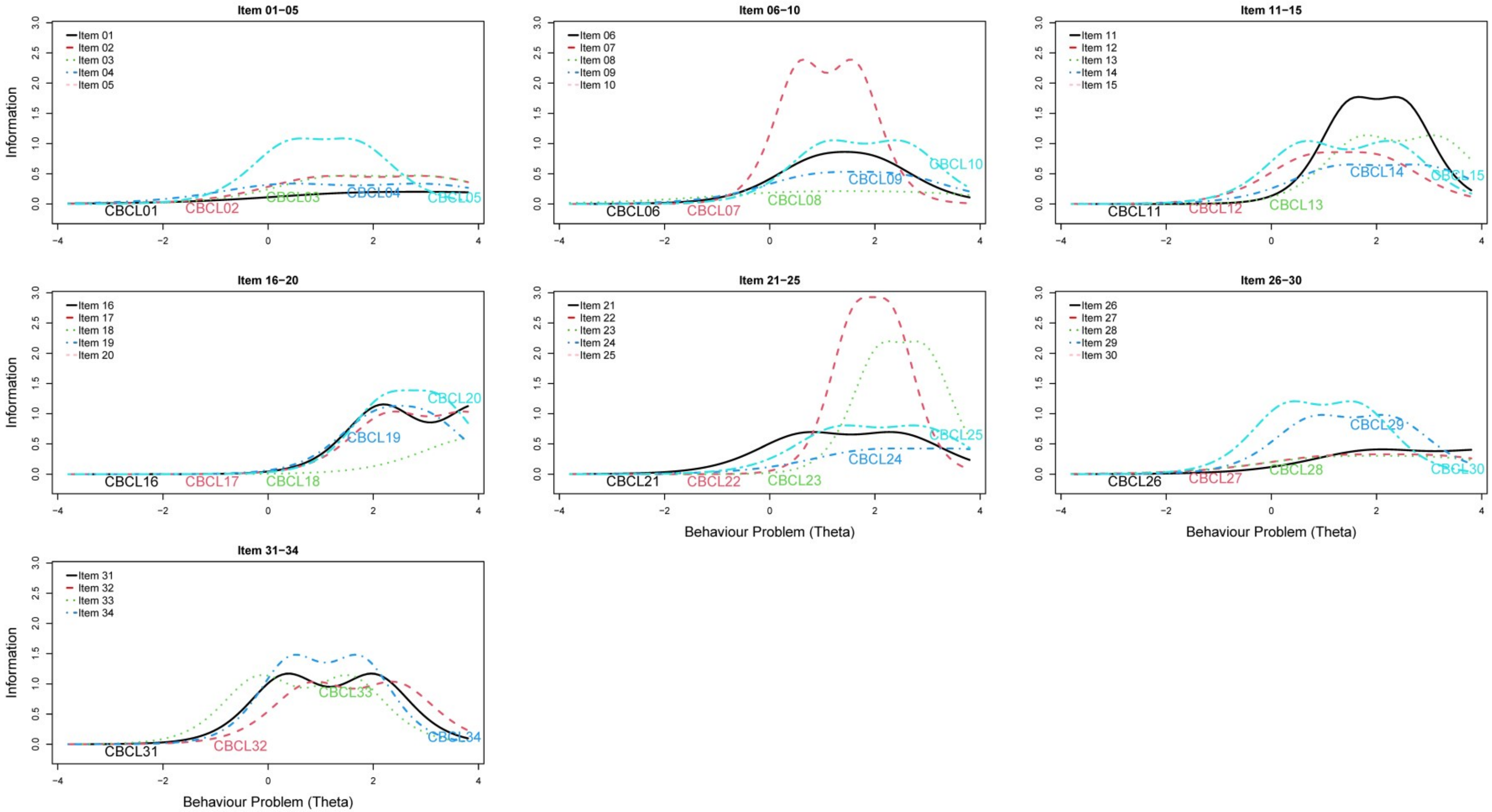

eFigure 8. Item information curves of the 34-item pool at T3 (Exploratory set, $N = 893$).

Abbreviations: *CBCL* Child Behavior Checklist.

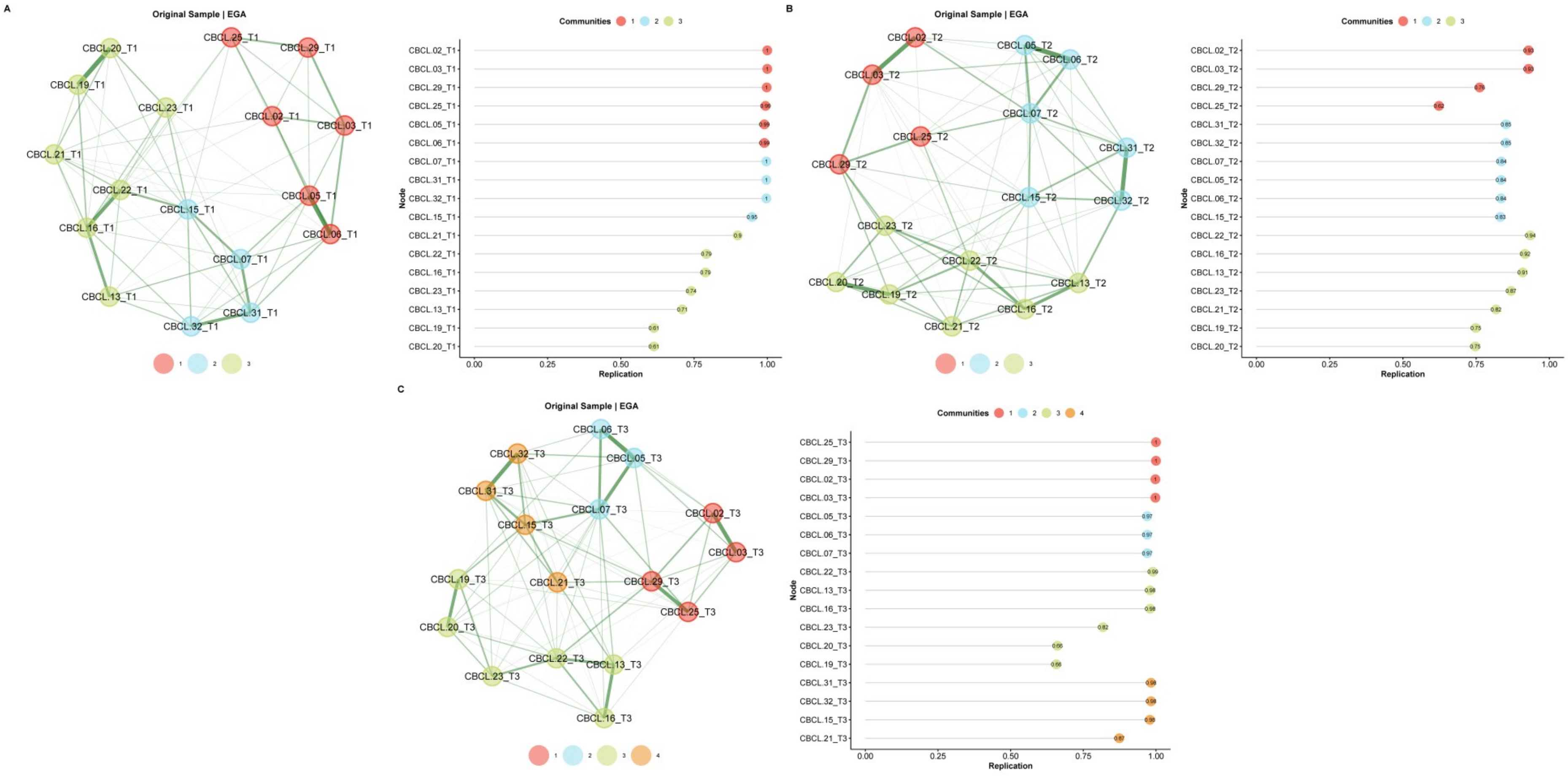

eFigure 9. Item clusters and stability of the 17-item pool at A) T1, B) T2, and C) T3 (Exploratory set, $N = 893$).

The overall output stabilities of the 17-item pool structures were valued at only 48.1%, 48.5%, and 65.3% at T1, T2, and T3, respectively.

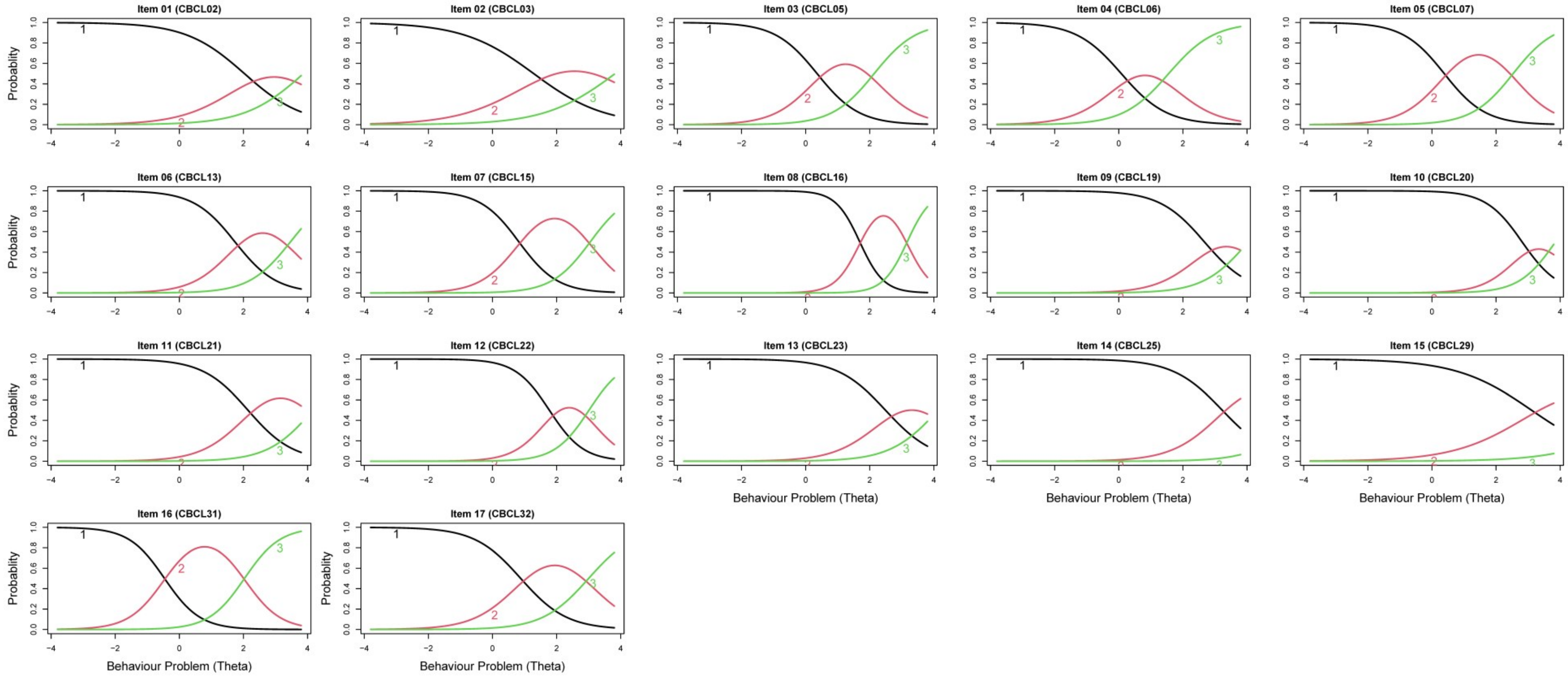

eFigure 10. Item characteristics of the 17-item pool at T1 (Exploratory set, $N = 893$).

Abbreviations: *CBCL* Child Behavior Checklist.

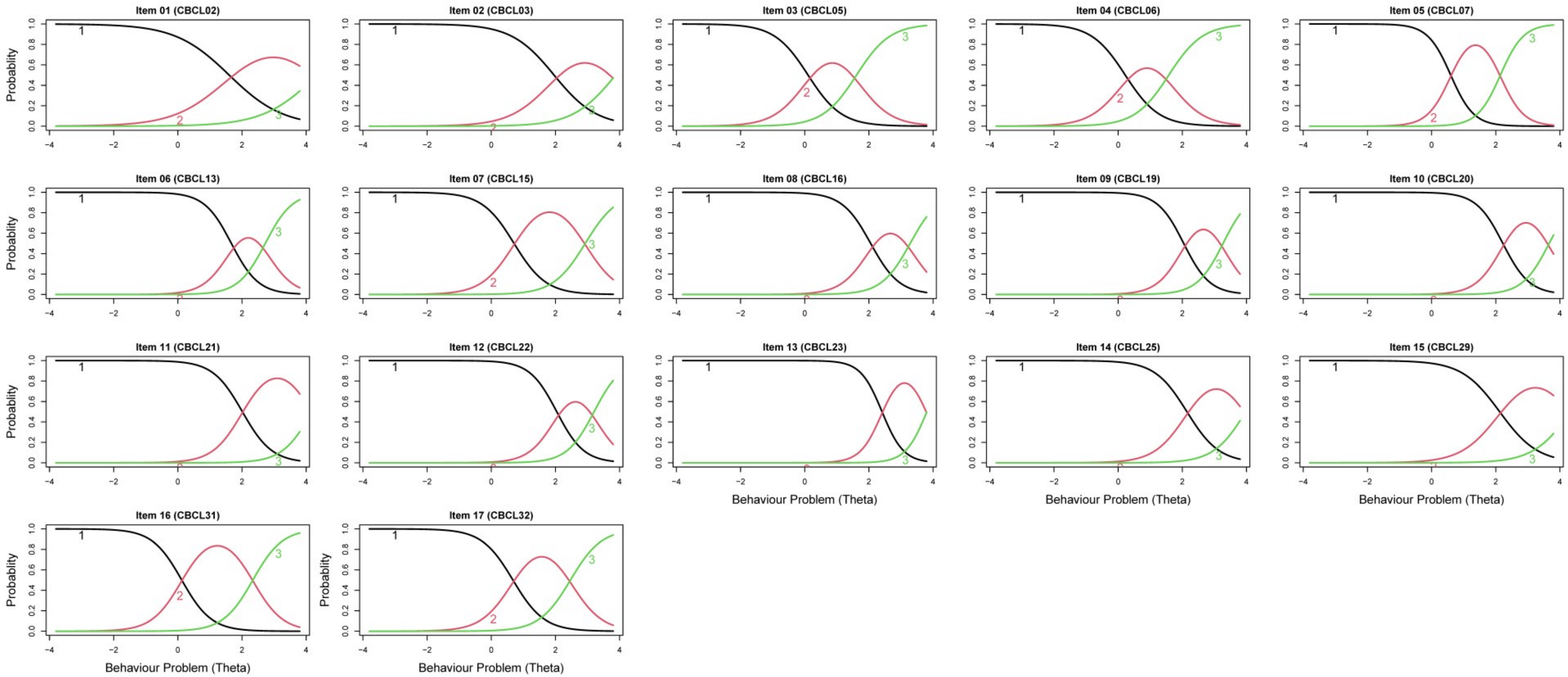

eFigure 11. Item characteristics of the 17-item pool at T2 (Exploratory set, $N = 893$).

Abbreviations: *CBCL* Child Behavior Checklist.

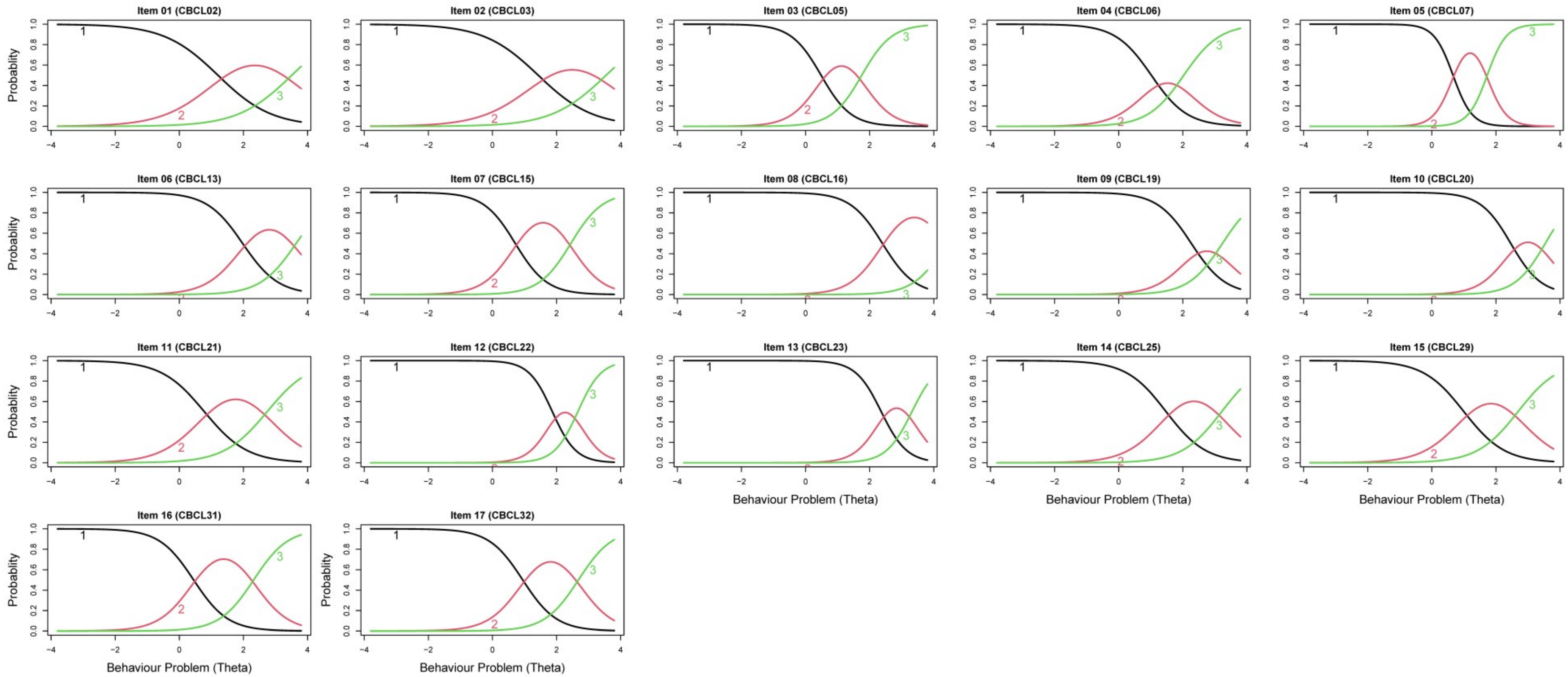

eFigure 12. Item characteristics of the 17-item pool at T3 (Exploratory set, *N* = 893).

Abbreviations: *CBCL* Child Behavior Checklist.

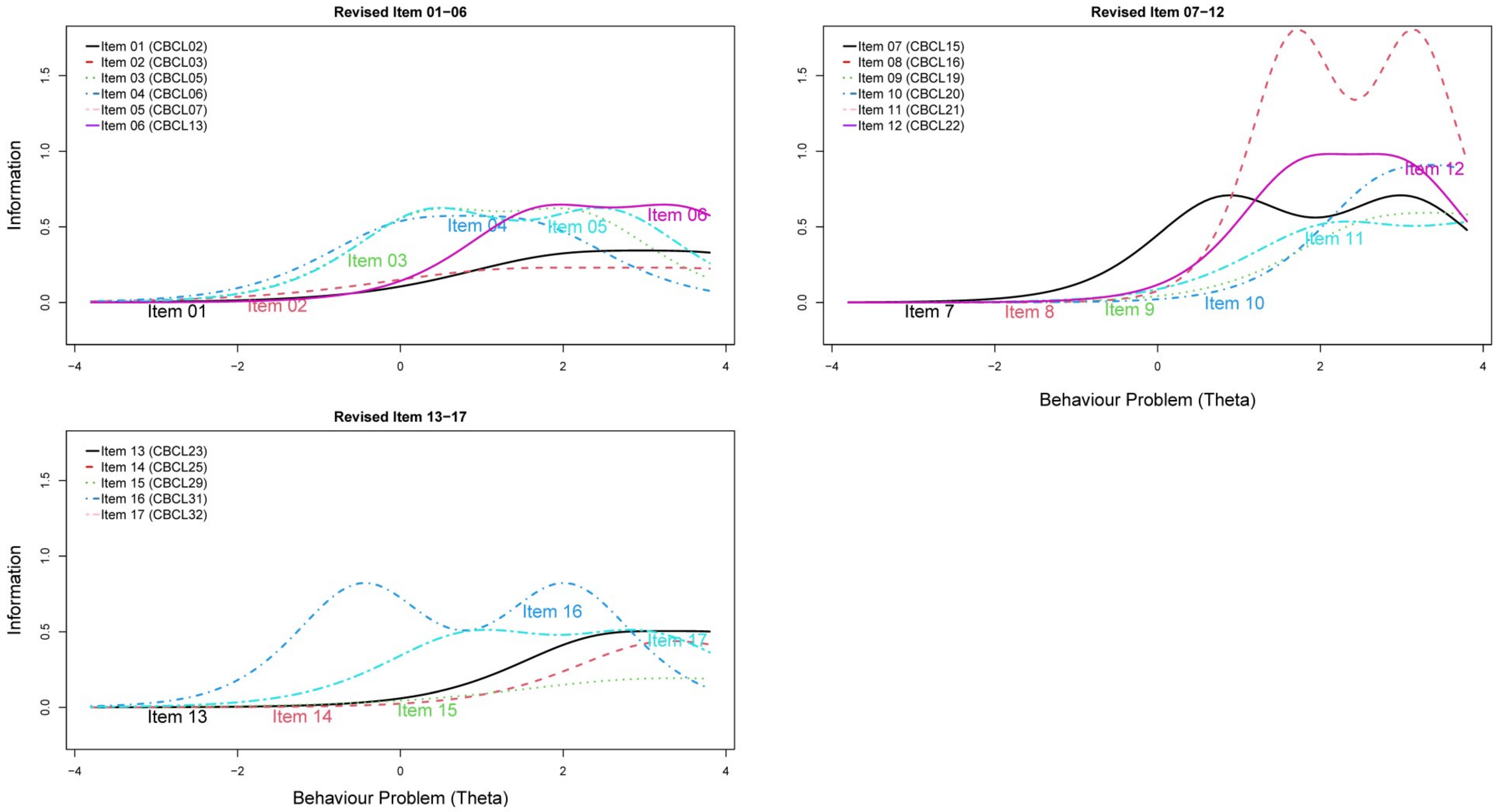

eFigure 13. Item information curves of the 17-item pool at T1 (Exploratory set, $N = 893$).

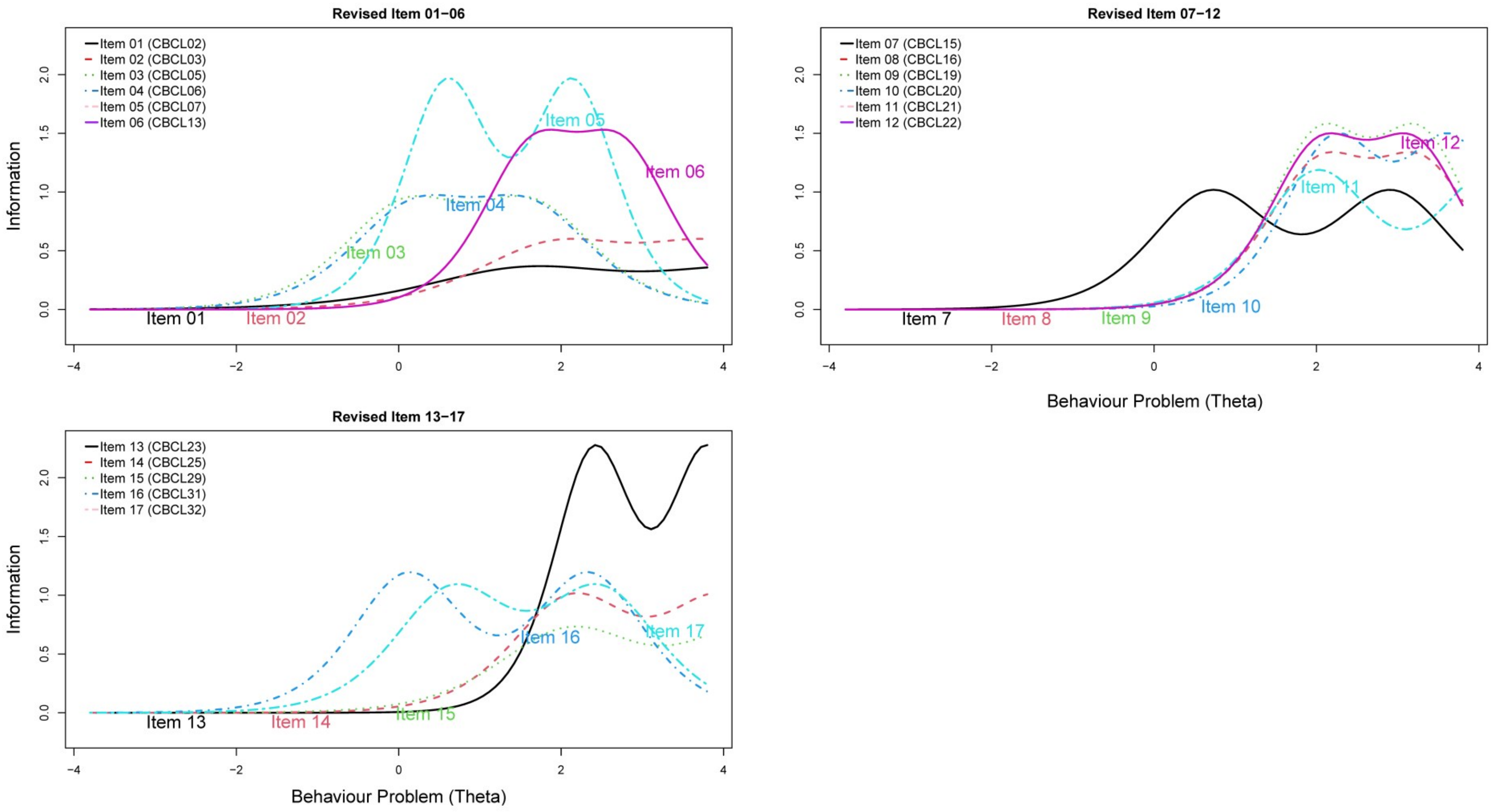

eFigure 14. Item information curves of the 17-item pool at T2 (Exploratory set, $N = 893$).

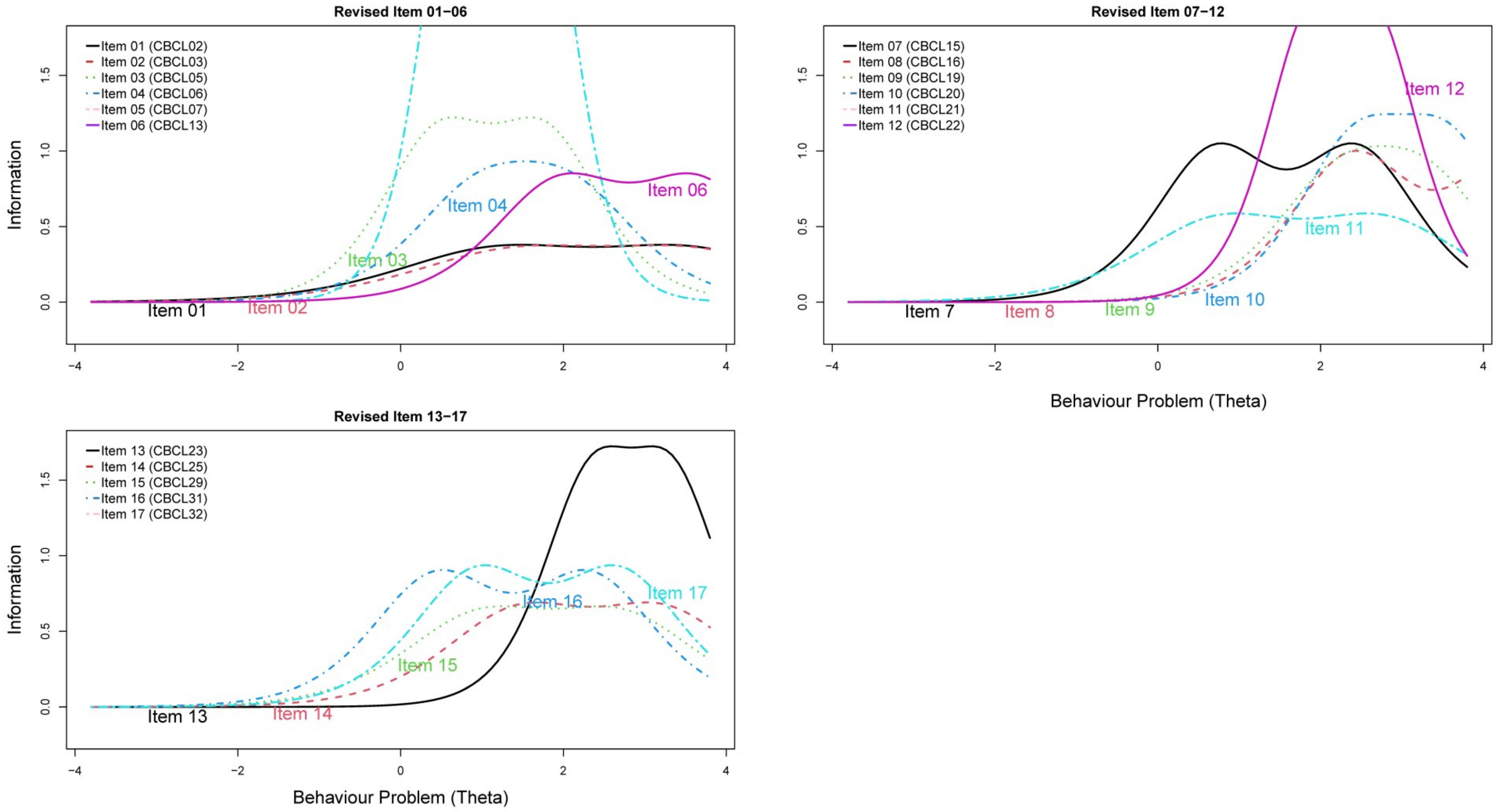

eFigure 15. Item information curves of the 17-item pool at T3 (Exploratory set, N = 893).

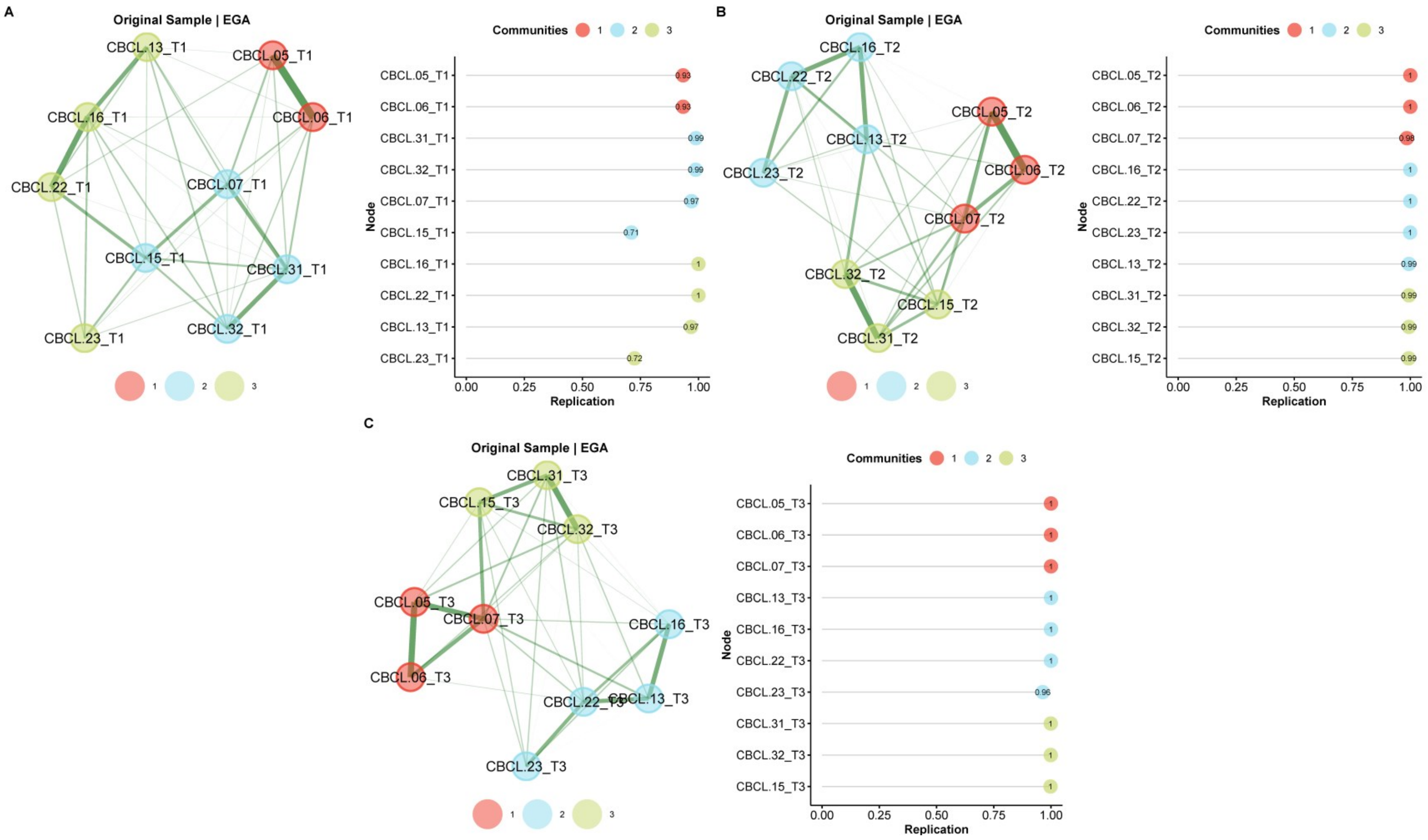

Figure 16. Visualized structural network of the second round revised CBCL (Exploratory data, $N = 893$).

Note: The thickness of the line indicates the strength of the edge, with thicker lines indicating a stronger link.

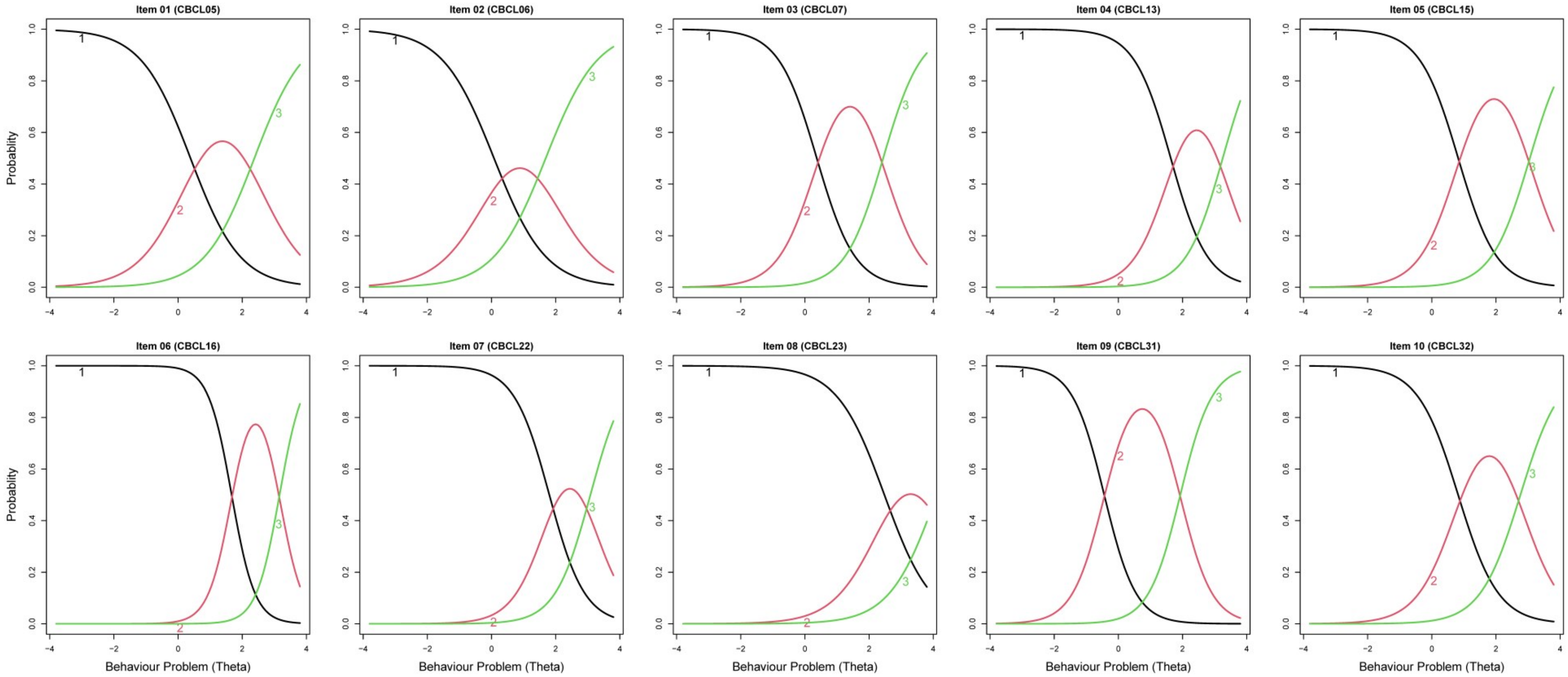

eFigure 17. Item characteristics of the 10-item pool at T1 (Exploratory set, $N = 893$).

Abbreviations: *CBCL* Child Behavior Checklist.

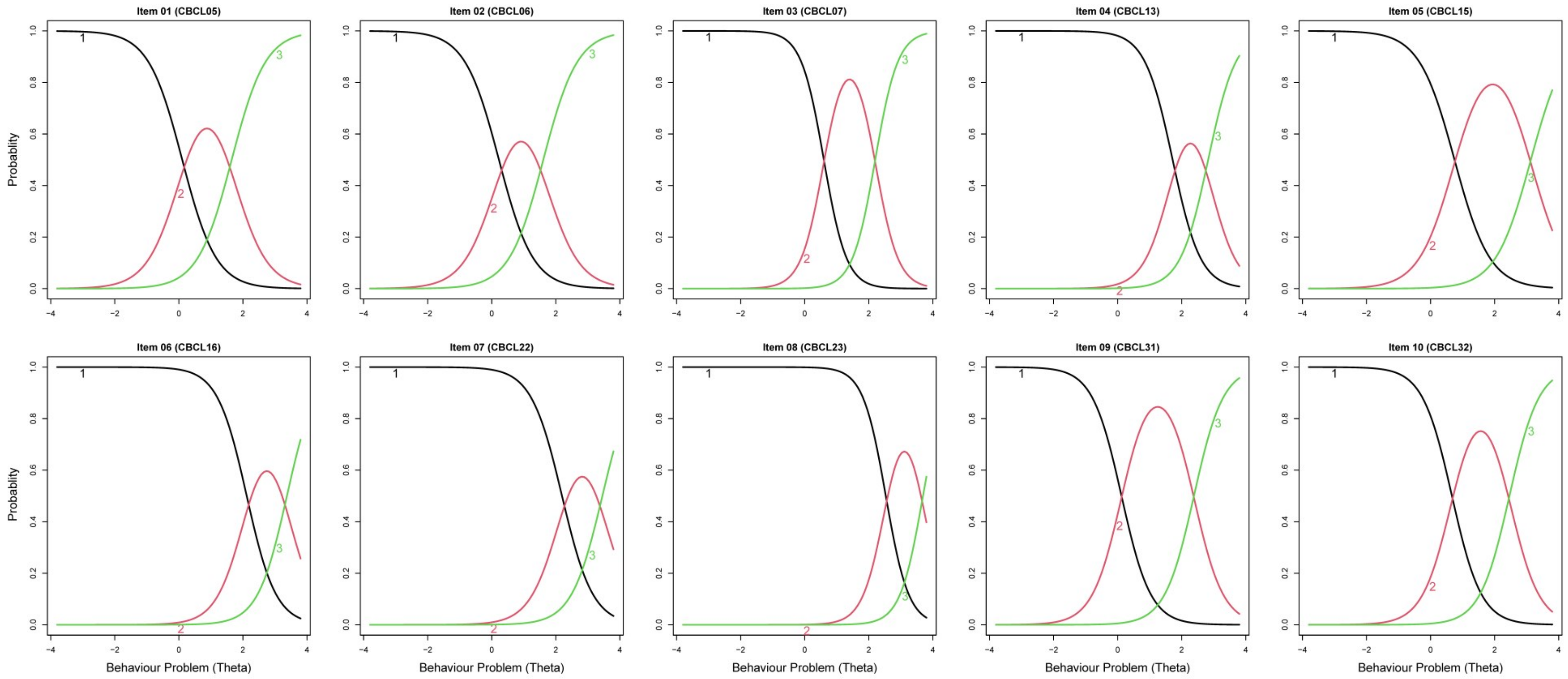

eFigure 18. Item characteristics of the 10-item pool at T2 (Exploratory set, *N* = 893).

Abbreviations: *CBCL* Child Behavior Checklist.

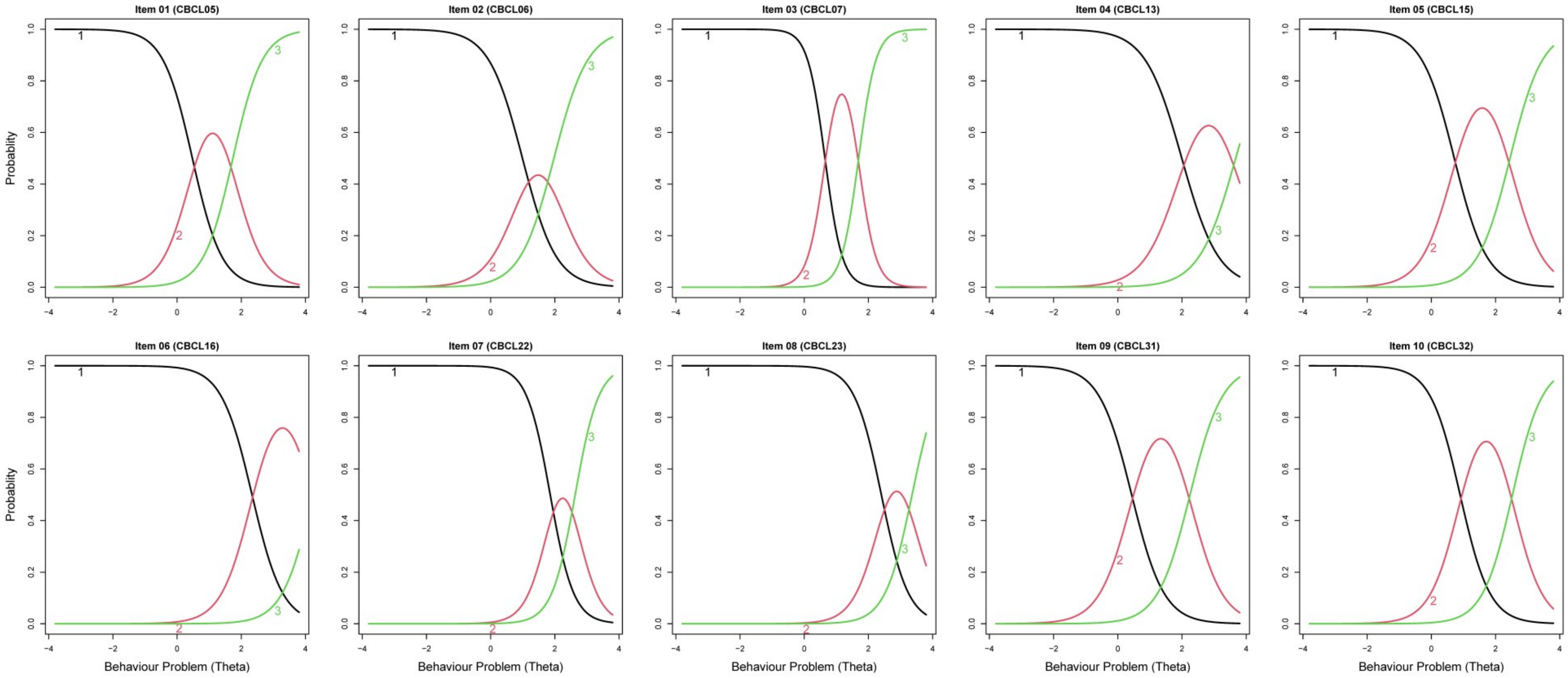

eFigure 19. Item characteristics of the 10-item pool at T3 (Exploratory set, N = 893).

Abbreviations: *CBCL* Child Behavior Checklist.

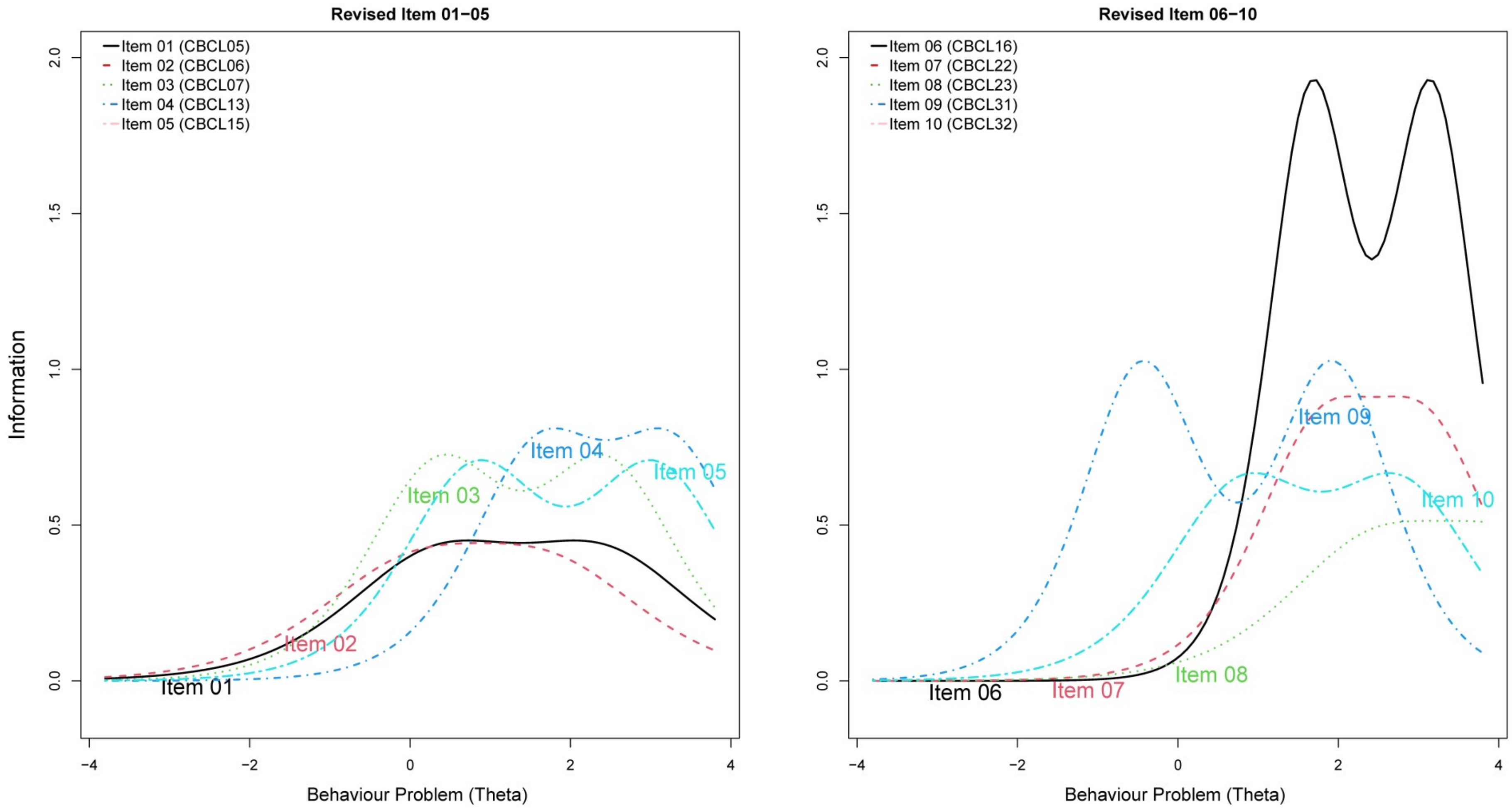

eFigure 20. Item information curves of the 10-item pool at T1 (Exploratory set, $N = 893$).

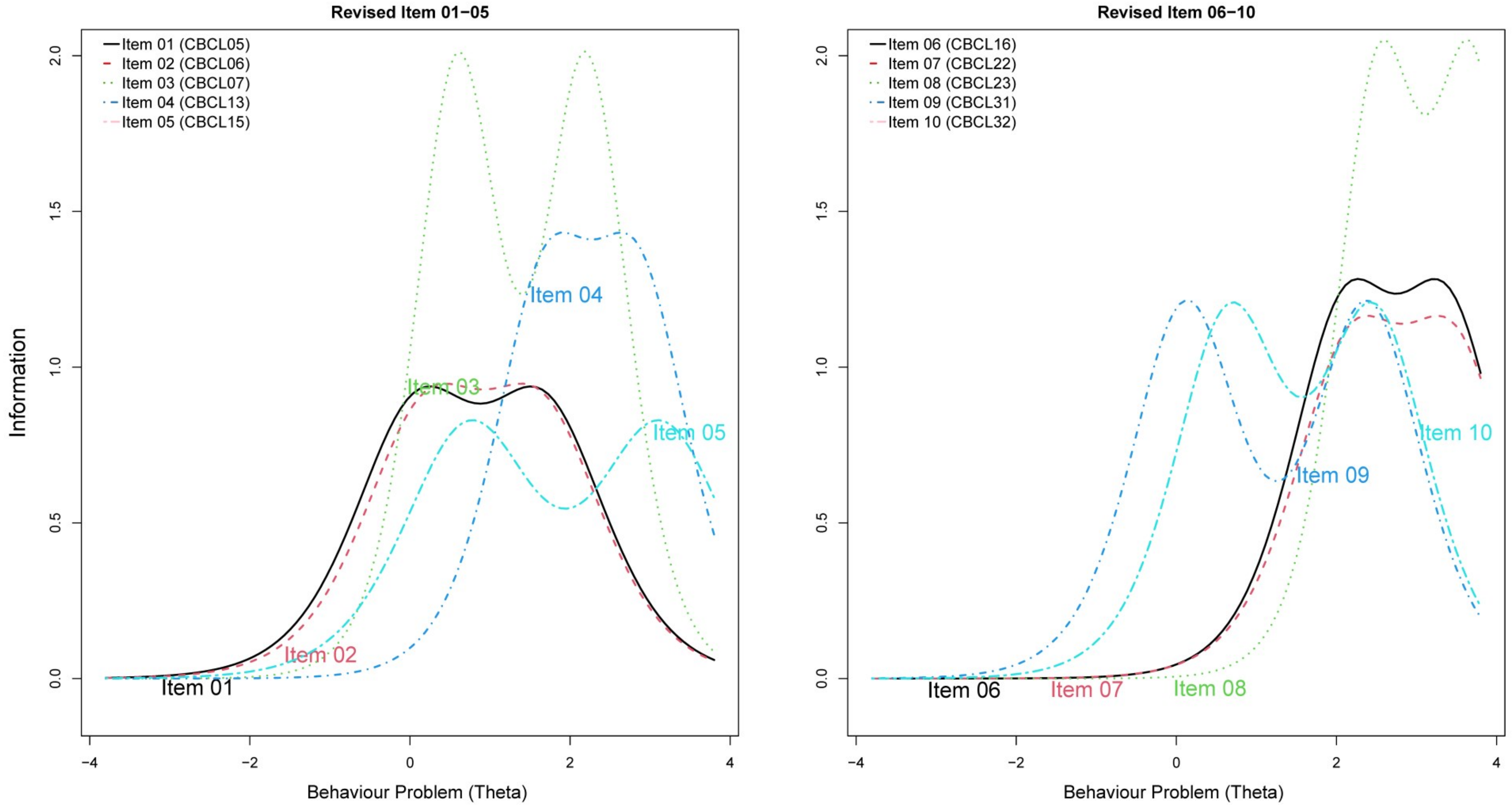

eFigure 21. Item information curves of the 10-item pool at T2 (Exploratory set, $N = 893$).

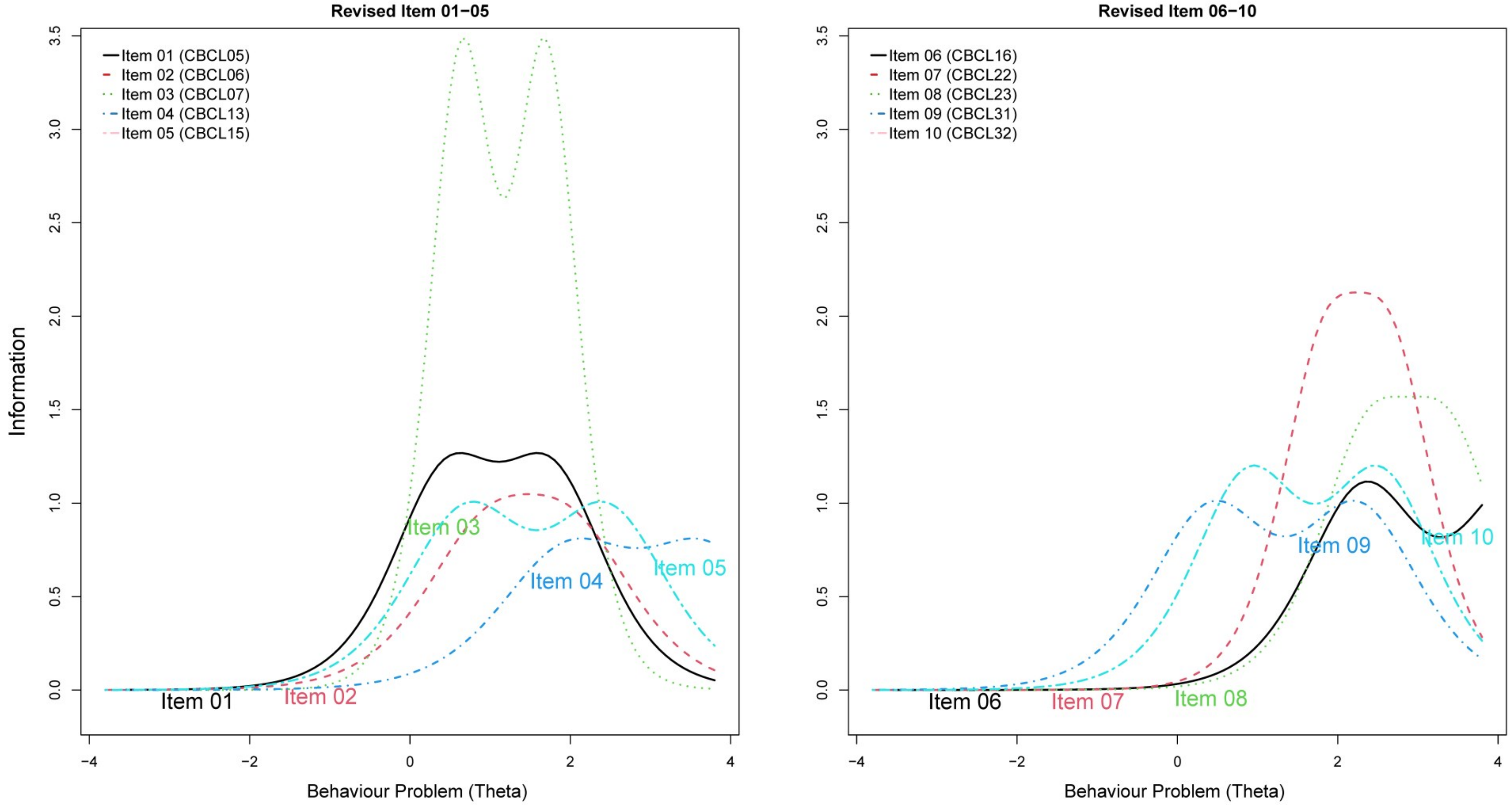

eFigure 22. Item information curves of the 10-item pool at T3 (Exploratory set, *N* = 893).
