# Supplementary material for: Measurement properties of the Child Behavior Checklist-10: an ultra-brief screening updated in longitudinal cohort
Source: Front Psychiatry. 2026 Apr 30;17:1767665. doi: 10.3389/fpsyt.2026.1767665 (PMC13171534; doi:10.3389/fpsyt.2026.1767665)
Supplement: Supplementary file 2 [file DataSheet2.docx]

**Supplement 2. eTables**

**Index**

**eTable 1.** Item removal protocol.

**eTable 2.** IRT parameters of original 34-item version at T1, T2, and T3 (Exploratory set, *N* = 893).

**eTable 3.** Reduction reasons for the first round (Exploratory set, *N* = 893).

**eTable 4.** IRT parameters of revised 17-item version at T1, T2, and T3 (Exploratory set, *N* = 893).

**eTable 5.** Reduction reasons for the second round (Exploratory set, *N* = 893).

**eTable 6.** IRT parameters of revised 10-item version at T1, T2, and T3 (Exploratory set, *N* = 893).

**eTable 7.** Various structural solutions and item versions of the CBCL.

**eTable 8.** Goodness-of-fit indices of different CBCL models (Confirmatory data, *N* = 893).

**eTable 9.** IRT parameters of the final 10-item version at T1, T2, and T3 (Full set, *N* = 1786).

**eTable 10.** Internal consistency of the revised 10-item CBCL (Full data, *N* = 1786).

**eTable 1.** Item removal protocol.

| **Protocol Phase** | **EGA Criteria** | **GRM Criteria** |
| --- | --- | --- |
| Initial Round | One of the two that was met should be considered for removal:  1. Item Stability: Value ≤ 0.650 (×) more than **two** time points;  2. Cluster Mixing Pattern (×) more than **two** time points. | Two of the three that were met should be considered for removal:  1. Item Discrimination^*^: 1) Slope ≤ 0.650 (×) at **any** time point; 2) Slope ≤ 1.350 (△) more than **two** time points;  2. Item Characteristics: Unideal Curves (×) more than **two** time points;  3. Item Information: Low Information (×) more than **two** time points. |
| Subsequent Round(s) | One of the two that was met should be considered for removal:  1. Item Stability^*^: 1) Value ≤ 0.650 (×) at **any** time point; 2) Value was lower than other items within the same factor (△) more than **two** time points;  2. Cluster Mixing Pattern (×) at **any** time point. | Two of the three that were met should be considered for removal:  1. Item Discrimination^*^: 1) Slope ≤ 0.650 (×) at **any** time point; 2) Slope ≤ 1.350 (△) more than **two** time points;  2. Item Characteristics^*^: 1) Unideal Curves (×) at **any** time point; 2) Relatively Unideal Curves (△) more than **two** time points;  3. Item Information: Low Information (×) more than **two** time points. |

^*^One of the two should consider for this criterion is met.

Item stability was considered unstable when value was lower than 0.651 (refers to: https://doi.org/10.3390/psych3030032).

Abbreviations: *EGA* Exploratory Graph Analysis, *GRM* Graded Response Model.

**eTable 2.** IRT parameters of original 34-item version at T1, T2, and T3 (Exploratory set, *N* = 893).

| **IP NO.** | **Ori NO.** | **Content** | **T1** | | | |  | **T2** | | | |  | **T3** | | | |
| --- | --- | --- | --- | --- | --- | --- | --- | --- | --- | --- | --- | --- | --- | --- | --- | --- |
|  |  |  | **a** | **b_1_** | **b_2_** | **Info** |  | **a** | **b_1_** | **b_2_** | **Info** |  | **a** | **b_1_** | **b_2_** | **Info** |
| **1** | **11** | Youth clings to adults or too dependent | 0.606 | 0.683 | 3.282 | 0.884 |  | 0.849 | 1.057 | 3.724 | 1.406 |  | 0.798 | 1.826 | 4.196 | 1.299 |
| **2** | **45** | Youth is nervous, highstrung, or tense | 1.120 | 2.000 | 3.804 | 1.764 |  | 1.225 | 1.577 | 4.300 | 2.234 |  | 1.291 | 1.140 | 3.296 | 2.296 |
| **3** | **50** | Youth is too fearful or anxious | 1.011 | 1.195 | 3.528 | 1.666 |  | 1.577 | 1.919 | 3.813 | 2.794 |  | 1.268 | 1.354 | 3.346 | 2.236 |
| **4** | **112** | Youth worries | 0.494 | 1.623 | 6.581 | 0.747 |  | 0.759 | 1.142 | 4.836 | 1.322 |  | 1.096 | 0.419 | 3.218 | 2.024 |
| **5** | **8** | Youth can't concentrate, can't pay attention for long | 1.440 | 0.368 | 2.212 | 2.457 |  | 1.614 | 0.109 | 1.767 | 2.778 |  | 1.922 | 0.508 | 1.815 | 3.383 |
| **6** | **10** | Youth can't sit still, is restless or hyperactive | 1.427 | 0.075 | 1.547 | 2.267 |  | 1.616 | 0.238 | 1.718 | 2.726 |  | 1.657 | 1.049 | 2.085 | 2.606 |
| **7** | **41** | Youth is impulsive or acts without thinking | 1.524 | 0.391 | 2.559 | 2.760 |  | 2.549 | 0.611 | 2.233 | 4.908 |  | 2.969 | 0.679 | 1.750 | 5.417 |
| **8** | **93** | Youth talks too much | 0.745 | -1.800 | 0.838 | 1.170 |  | 1.011 | 0.218 | 2.616 | 1.712 |  | 0.829 | 0.496 | 2.828 | 1.347 |
| **9** | **104** | Youth is unusually loud | 1.200 | -0.036 | 1.899 | 1.970 |  | 1.440 | 1.266 | 2.996 | 2.482 |  | 1.324 | 1.007 | 2.559 | 2.185 |
| **10** | **16** | Youth is cruel, bullies, or shows meanness to others | 1.555 | 1.038 | 3.012 | 2.731 |  | 2.342 | 1.328 | 2.534 | 4.041 |  | 1.910 | 1.216 | 2.725 | 3.506 |
| **11** | **21** | Youth destroys things belonging to family or others | 1.552 | 1.206 | 2.632 | 2.516 |  | 2.238 | 1.190 | 2.680 | 4.089 |  | 2.498 | 1.641 | 2.705 | 4.378 |
| **12** | **26** | Youth doesn't seem to feel guilty after misbehaving | 0.662 | 0.939 | 4.038 | 1.037 |  | 1.474 | 1.207 | 3.174 | 2.652 |  | 1.690 | 0.848 | 2.170 | 2.866 |
| **13** | **37** | Youth gets in many fights | 1.588 | 1.717 | 3.414 | 2.686 |  | 2.321 | 1.693 | 2.769 | 3.888 |  | 1.933 | 1.893 | 3.459 | 3.714 |
| **14** | **39** | Youth hangs around with others who get in trouble | 1.027 | 2.367 | 4.745 | 1.710 |  | 1.623 | 1.828 | 3.737 | 2.968 |  | 1.475 | 1.423 | 3.188 | 2.665 |
| **15** | **43** | Youth lies or cheats | 1.593 | 0.845 | 3.123 | 2.944 |  | 2.092 | 0.699 | 2.893 | 4.083 |  | 1.921 | 0.727 | 2.469 | 3.652 |
| **16** | **57** | Youth physically attacks people | 2.669 | 1.690 | 3.144 | 4.789 |  | 2.307 | 2.053 | 3.273 | 3.999 |  | 2.003 | 2.361 | 4.322 | 4.001 |
| **17** | **67** | Youth runs away from home | 0.678 | 7.267 | 8.727 | 0.675 |  | 1.921 | 3.106 | 4.224 | 3.148 |  | 1.860 | 2.511 | 4.128 | 3.532 |
| **18** | **72** | Youth sets fires | 1.641 | 3.524 | 4.217 | 2.226 |  | 1.542 | 3.655 | 4.743 | 2.355 |  | 1.520 | 3.887 | 5.200 | 2.353 |
| **19** | **81** | Youth steals at home | 1.406 | 2.702 | 4.083 | 2.154 |  | 2.225 | 2.143 | 3.470 | 3.978 |  | 1.820 | 2.279 | 3.244 | 3.018 |
| **20** | **82** | Youth steals outside the home | 1.662 | 2.906 | 4.001 | 2.525 |  | 2.240 | 2.311 | 3.910 | 4.180 |  | 2.052 | 2.463 | 3.530 | 3.623 |
| **21** | **90** | Youth swears or uses obscene language | 1.459 | 2.108 | 4.072 | 2.513 |  | 2.169 | 2.032 | 4.489 | 4.323 |  | 1.581 | 0.756 | 2.614 | 2.858 |
| **22** | **97** | Youth threatens people | 2.004 | 1.725 | 2.911 | 3.277 |  | 2.621 | 1.990 | 3.085 | 4.788 |  | 3.060 | 1.777 | 2.516 | 5.287 |
| **23** | **106** | Youth vandalizes | 1.479 | 2.344 | 3.858 | 2.385 |  | 2.729 | 2.514 | 4.226 | 5.296 |  | 2.731 | 2.260 | 3.169 | 4.779 |
| **24** | **14** | Youth cries a lot | 0.918 | 1.098 | 2.703 | 1.315 |  | 1.019 | 1.800 | 3.996 | 1.697 |  | 1.209 | 2.056 | 4.016 | 2.070 |
| **25** | **35** | Youth feels worthless or inferior | 1.320 | 3.192 | 5.776 | 2.367 |  | 1.978 | 2.170 | 4.145 | 3.697 |  | 1.700 | 1.429 | 3.088 | 3.028 |
| **26** | **52** | Youth feels too guilty | 0.910 | 2.806 | 5.761 | 1.518 |  | 1.202 | 2.980 | 5.915 | 2.248 |  | 1.189 | 2.099 | 4.615 | 2.216 |
| **27** | **100** | Youth has trouble sleeping | 0.672 | 1.690 | 4.406 | 1.011 |  | 1.317 | 2.111 | 3.782 | 2.191 |  | 1.032 | 1.306 | 3.365 | 1.744 |
| **28** | **102** | Youth is underactive, slow moving, or lacks energy | 0.546 | 5.112 | 8.124 | 0.673 |  | 1.141 | 2.692 | 4.764 | 1.916 |  | 0.994 | 1.201 | 3.274 | 1.652 |
| **29** | **103** | Youth is unhappy, sad, or depressed | 0.890 | 3.014 | 6.506 | 1.519 |  | 1.854 | 2.042 | 4.296 | 3.603 |  | 1.847 | 0.925 | 2.440 | 3.339 |
| **30** | **3** | Youth argues a lot | 1.194 | -0.884 | 1.246 | 2.014 |  | 1.508 | -0.009 | 2.034 | 2.748 |  | 2.083 | 0.401 | 1.778 | 3.728 |
| **31** | **22** | Youth is disobedient at home | 1.763 | -0.465 | 2.051 | 3.353 |  | 2.164 | 0.124 | 2.365 | 4.226 |  | 2.065 | 0.433 | 2.193 | 3.943 |
| **32** | **23** | Youth is disobedient at school | 1.225 | 0.953 | 3.270 | 2.120 |  | 1.862 | 0.703 | 2.603 | 3.488 |  | 1.910 | 0.936 | 2.629 | 3.605 |
| **33** | **86** | Youth is stubborn, sullen, or irritable | 1.274 | 0.015 | 2.208 | 2.197 |  | 1.861 | 0.666 | 2.522 | 3.484 |  | 2.049 | -0.082 | 1.682 | 3.903 |
| **34** | **95** | Youth has temper tantrums or a hot temper | 1.228 | -0.053 | 2.060 | 2.084 |  | 2.132 | 0.791 | 2.104 | 3.775 |  | 2.341 | 0.542 | 1.891 | 4.254 |

First-to-second eigenvalue ratio = 2.80–6.41 across T1–T3; C_2_ statistic fit indices: CFI = 0.872–0.905, TLI = 0.853–0.890, RMSEA = 0.070–0.088; Yen's Q3 mean residual correlation = -0.02 to -0.03, with 13–30 item pairs exceeding the |Q3| > 0.20 threshold across timepoints.

Abbreviations**:** *IRT* Item Response Theory, *IP NO.* item pool number, *Ori NO.* original item number, *Info* amount of information between -4 SD and +4 SD around the latent trait mean, *T1–T3* timepoints 1–3, *a* discrimination/slope parameter, *b_1_–b_2_* difficulty parameters.

**eTable 3.** Reduction reasons for the first round (Exploratory set, *N* = 893).

| **IP NO.** | **Ori NO.** | **Reduction Reason(s)** | | | | | | | | | | | | | | |
| --- | --- | --- | --- | --- | --- | --- | --- | --- | --- | --- | --- | --- | --- | --- | --- | --- |
|  |  | **EGA** | | | | | | **IRT** | | | | | | | | |
|  |  | **Cluster** | | | **Stability** | | | **Characteristics** | | | **Discrimination** | | | **Information** | | |
|  |  | **T1** | **T2** | **T3** | **T1** | **T2** | **T3** | **T1** | **T2** | **T3** | **T1** | **T2** | **T3** | **T1** | **T2** | **T3** |
| **1^#^** | **11** |  | **△** |  |  | **×** |  | **×** | **×** | **×** | **×** | **△** | **△** | **×** | **×** | **×** |
| **2** | **45** |  |  |  |  |  |  | **△** | **△** | **△** | **△** | **△** | **△** |  |  | **△** |
| **3** | **50** |  |  |  |  |  |  | **△** | **△** | **△** | **△** |  | **△** |  |  | **△** |
| **4^#^** | **112** | **×** |  |  | **×** |  |  | **×** | **×** | **△** | **×** | **△** | **△** | **×** | **×** | **×** |
| **5** | **8** |  |  | **△** |  |  |  |  |  |  |  |  |  |  |  |  |
| **6** | **10** |  |  | **△** |  |  |  |  |  | **△** |  |  |  |  |  |  |
| **7** | **41** |  |  | **△** |  |  |  |  |  |  |  |  |  |  |  |  |
| **8^#*^** | **93** |  |  |  |  | **×** | **×** | **△** | **×** | **×** | **△** | **△** | **△** | **×** | **×** | **×** |
| **9^*^** | **104** |  |  |  |  | **×** | **×** | **△** |  | **△** | **△** |  | **△** | **△** | **△** | **△** |
| **10^*^** | **16** |  |  | **×** | **×** |  | **×** |  |  |  |  |  |  |  | **△** |  |
| **11^*^** | **21** |  |  |  | **×** | **×** |  |  |  |  |  |  |  |  |  |  |
| **12^*^** | **26** |  | **△** | **×** |  | **×** | **×** | **×** | **△** | **△** | **△** |  |  | **×** | **×** | **×** |
| **13** | **37** | **△** |  |  | **×** |  |  | **△** |  |  |  |  |  |  |  |  |
| **14^*^** | **39** | **△** |  |  | **×** | **×** |  | **×** | **△** |  | **△** |  |  | **×** | **×** | **×** |
| **15** | **43** |  |  |  | **△** | **×** |  |  |  |  |  |  |  |  |  | **△** |
| **16** | **57** |  |  |  |  |  |  |  |  | **△** |  |  |  |  |  |  |
| **17^#^** | **67** | **×** | **△** |  |  | **×** |  | **×** | **×** | **△** | **△** |  |  | **×** | **×** | **△** |
| **18^#*^** | **72** | **△** |  |  | **×** | **×** |  | **×** | **×** | **×** |  |  |  | **△** | **×** | **×** |
| **19** | **81** |  |  |  |  |  |  | **△** |  | **△** |  |  |  |  |  |  |
| **20** | **82** |  |  |  |  |  |  | **△** | **△** |  |  |  |  |  |  |  |
| **21** | **90** |  |  |  |  |  |  | **△** | **△** |  |  |  |  |  |  | **×** |
| **22** | **97** |  |  |  |  |  |  |  |  |  |  |  |  |  |  |  |
| **23** | **106** |  |  |  | **×** |  |  | **△** | **△** |  |  |  |  |  |  |  |
| **24^#^** | **14** |  | **△** |  |  | **×** |  | **×** | **×** | **△** | **△** | **△** | **△** | **△** | **×** | **×** |
| **25** | **35** |  |  |  |  |  |  | **×** | **△** |  | **△** |  |  |  |  | **△** |
| **26^#^** | **52** | **×** |  |  |  |  |  | **×** | **×** | **△** | **△** | **△** | **△** | **△** | **×** | **△** |
| **27^#^** | **100** | **×** |  |  |  |  |  | **×** | **△** | **×** | **△** | **△** | **△** | **×** | **△** | **×** |
| **28^#^** | **102** | **△** |  |  | **△** |  |  | **×** | **×** | **×** | **×** | **△** | **△** | **×** | **×** | **×** |
| **29** | **103** |  | **△** |  |  |  |  | **×** | **△** |  | **△** |  |  | **△** |  |  |
| **30^*^** | **3** |  |  |  |  | **×** | **×** |  |  |  | **△** |  |  |  |  |  |
| **31** | **22** |  |  |  |  | **×** |  |  |  |  |  |  |  |  |  |  |
| **32** | **23** |  |  |  |  | **×** |  |  |  |  | **△** |  |  | **×** |  |  |
| **33^*^** | **86** |  |  | **×** |  | **×** | **×** |  |  |  | **△** |  |  | **×** |  |  |
| **34^*^** | **95** |  |  | **×** |  | **×** | **×** |  |  |  | **△** |  |  | **×** |  |  |

Green, brown, and blue background colors stand for parameters at time points one, two, and three, respectively. Red mark stands for this removal criteria was met.

Abbreviations: *IP NO.* item pool number, *Ori NO.* original item number, ^*^ Items have met the EGA removal standard, *^#^* Items have met the IRT removal standard, *IRT* Item Response Theory, *Info* amount of information between -4 SD and +4 SD around the latent trait mean, *T1–T3* timepoints 1–3, *a* discrimination/slope parameter, *b_1_–b_2_* difficulty parameters.

**eTable 4.** IRT parameters of revised 17-item version at T1, T2, and T3 (Exploratory set, *N* = 893).

| **IP NO.** | **Cur NO.** | **Content** | **T1** | | | |  | **T2** | | | |  | **T3** | | | |
| --- | --- | --- | --- | --- | --- | --- | --- | --- | --- | --- | --- | --- | --- | --- | --- | --- |
|  |  |  | **a** | **b_1_** | **b_2_** | **Info** |  | **a** | **b_1_** | **b_2_** | **Info** |  | **a** | **b_1_** | **b_2_** | **Info** |
| **2** | **1** | Youth is nervous, highstrung, or tense | 1.089 | 2.037 | 3.890 | 1.744 |  | 1.180 | 1.624 | 4.400 | 2.156 |  | 1.204 | 1.192 | 3.473 | 2.068 |
| **3** | **2** | Youth is too fearful or anxious | 0.908 | 1.289 | 3.844 | 1.509 |  | 1.493 | 1.987 | 3.932 | 2.650 |  | 1.192 | 1.409 | 3.500 | 1.986 |
| **5** | **3** | Youth can't concentrate, can't pay attention for long | 1.520 | 0.357 | 2.145 | 2.641 |  | 1.881 | 0.110 | 1.646 | 3.359 |  | 2.181 | 0.477 | 1.729 | 3.696 |
| **6** | **4** | Youth can't sit still, is restless or hyperactive | 1.414 | 0.077 | 1.562 | 2.287 |  | 1.858 | 0.229 | 1.613 | 3.238 |  | 1.811 | 1.005 | 2.006 | 2.759 |
| **7** | **5** | Youth is impulsive or acts without thinking | 1.553 | 0.383 | 2.534 | 2.826 |  | 2.741 | 0.607 | 2.191 | 5.322 |  | 3.412 | 0.653 | 1.712 | 6.251 |
| **13** | **6** | Youth gets in many fights | 1.556 | 1.735 | 3.460 | 2.678 |  | 2.281 | 1.715 | 2.809 | 4.013 |  | 1.799 | 1.967 | 3.625 | 3.191 |
| **15** | **7** | Youth lies or cheats | 1.664 | 0.825 | 3.048 | 3.090 |  | 1.975 | 0.724 | 2.988 | 3.849 |  | 2.046 | 0.705 | 2.413 | 3.709 |
| **16** | **8** | Youth physically attacks people | 2.685 | 1.686 | 3.149 | 5.005 |  | 2.218 | 2.094 | 3.349 | 3.892 |  | 1.990 | 2.381 | 4.362 | 3.726 |
| **19** | **9** | Youth steals at home | 1.438 | 2.666 | 4.028 | 2.264 |  | 2.332 | 2.112 | 3.391 | 4.358 |  | 1.891 | 2.249 | 3.209 | 2.909 |
| **20** | **10** | Youth steals outside the home | 1.776 | 2.806 | 3.842 | 2.743 |  | 2.300 | 2.297 | 3.799 | 4.429 |  | 2.145 | 2.433 | 3.494 | 3.477 |
| **21** | **11** | Youth swears or uses obscene language | 1.420 | 2.144 | 4.171 | 2.496 |  | 2.071 | 2.080 | 4.326 | 4.195 |  | 1.497 | 0.775 | 2.716 | 2.623 |
| **22** | **12** | Youth threatens people | 1.893 | 1.771 | 3.004 | 3.126 |  | 2.265 | 2.107 | 3.317 | 4.118 |  | 2.763 | 1.848 | 2.629 | 4.427 |
| **23** | **13** | Youth vandalizes | 1.345 | 2.485 | 4.123 | 2.187 |  | 2.868 | 2.489 | 3.930 | 5.691 |  | 2.547 | 2.331 | 3.281 | 4.182 |
| **25** | **14** | Youth feels worthless or inferior | 1.307 | 3.219 | 5.831 | 2.372 |  | 1.963 | 2.185 | 4.064 | 3.692 |  | 1.631 | 1.463 | 3.174 | 2.805 |
| **29** | **15** | Youth is unhappy, sad, or depressed | 0.855 | 3.113 | 6.736 | 1.483 |  | 1.650 | 2.174 | 4.455 | 3.155 |  | 1.599 | 0.989 | 2.650 | 2.703 |
| **31** | **16** | Youth is disobedient at home | 1.805 | -0.462 | 2.033 | 3.465 |  | 2.136 | 0.127 | 2.391 | 4.224 |  | 1.896 | 0.441 | 2.292 | 3.447 |
| **32** | **17** | Youth is disobedient at school | 1.397 | 0.881 | 2.995 | 2.464 |  | 2.003 | 0.694 | 2.534 | 3.842 |  | 1.918 | 0.935 | 2.652 | 3.447 |

First-to-second eigenvalue ratio = 2.85–6.79 across T1–T3; C_2_ statistic fit indices: CFI = 0.890–0.940, TLI = 0.875–0.930, RMSEA = 0.060–0.080; Yen's Q3 mean residual correlation = -0.03 to -0.05, with 8–18 item pairs exceeding the |Q3| > 0.20 threshold across timepoints.

Abbreviations: *IRT* Item Response Theory, *IP NO.* item pool number, *Cur NO.* original item number, *Info* amount of information between -4 SD and +4 SD around the latent trait mean, *T1–T3* timepoints 1–3, *a* discrimination/slope parameter, *b_1_–b_2_* difficulty parameters.

**eTable 5.** Reduction reasons for the second round (Exploratory set, *N* = 893).

| **Initial**  **IP NO.** | **Current NO.** | **Reduction Reason(s)** | | | | | | | | | | | | | | |
| --- | --- | --- | --- | --- | --- | --- | --- | --- | --- | --- | --- | --- | --- | --- | --- | --- |
|  |  | **EGA** | | | | | | **IRT** | | | | | | | | |
|  |  | **Cluster** | | | **Stability** | | | **Characteristics** | | | **Discrimination** | | | **Information** | | |
|  |  | **T1** | **T2** | **T3** | **T1** | **T2** | **T3** | **T1** | **T2** | **T3** | **T1** | **T2** | **T3** | **T1** | **T2** | **T3** |
| **2^#^** | **1** |  |  |  |  |  |  | **△** | **△** | **△** | **△** | **△** | **△** | **×** | **×** | **×** |
| **3^#^** | **2** |  |  |  |  |  |  | **△** | **△** | **△** | **△** |  | **△** | **×** | **×** | **×** |
| **5** | **3** |  |  |  |  |  |  |  |  |  |  |  |  |  |  |  |
| **6** | **4** |  |  |  |  |  |  |  |  | **△** |  |  |  |  |  |  |
| **7** | **5** |  |  |  |  |  |  |  |  |  |  |  |  |  |  |  |
| **13** | **6** |  |  |  |  |  |  | **△** |  |  |  |  |  |  |  |  |
| **15** | **7** |  |  |  |  |  |  | **△** |  |  |  |  |  | **×** | **×** |  |
| **16** | **8** |  |  |  |  |  |  |  |  |  |  |  |  |  | **△** |  |
| **19^*^** | **9** |  |  |  | **△** | **△** | **△** | **△** |  |  |  |  |  | **×** |  | **△** |
| **20^*^** | **10** |  |  |  | **△** | **△** | **△** | **△** |  |  |  |  |  | **△** |  |  |
| **21^#^** | **11** |  |  |  |  |  | **△** | **△** | **△** |  |  |  |  | **×** | **×** | **×** |
| **22** | **12** |  |  |  |  |  |  |  |  |  |  |  |  |  |  |  |
| **23** | **13** |  |  |  |  |  | **△** | **△** | **△** |  | **△** |  |  | **△** |  |  |
| **25^#*^** | **14** |  |  |  |  | **×** |  | **×** | **△** |  | **△** |  |  | **×** | **×** | **×** |
| **29^*^** | **15** |  |  |  |  | **△** |  | **×** | **△** |  | **△** |  |  | **×** | **×** | **×** |
| **31** | **16** |  |  |  |  |  |  |  |  |  |  |  |  |  |  | **△** |
| **32** | **17** |  |  |  |  |  |  |  |  |  |  |  |  |  | **△** |  |

Green, brown, and blue background colors stand for parameters at time points one, two, and three, respectively. Red mark stands for this removal criteria was met.

Abbreviations: *IP NO.* item pool number, ^*^ Items have met the EGA removal standard, *^#^* Items have met the IRT removal standard, *IRT* Item Response Theory, *Info* amount of information between -4 SD and +4 SD around the latent trait mean, *T1–T3* timepoints 1–3, *a* discrimination/slope parameter, *b_1_–b_2_* difficulty parameters.

**eTable 6.** IRT parameters of revised 10-item version at T1, T2, and T3 (Exploratory set, *N* = 893).

| **IP NO.** | **Cur NO.** | **Content** | **T1** | | | |  | **T2** | | | |  | **T3** | | | |
| --- | --- | --- | --- | --- | --- | --- | --- | --- | --- | --- | --- | --- | --- | --- | --- | --- |
|  |  |  | **a** | **b_1_** | **b_2_** | **Info** |  | **a** | **b_1_** | **b_2_** | **Info** |  | **a** | **b_1_** | **b_2_** | **Info** |
| **5** | **1** | Youth can't concentrate, can't pay attention for long | 1.288 | 0.389 | 2.380 | 2.197 |  | 1.877 | 0.108 | 1.658 | 3.317 |  | 2.209 | 0.476 | 1.727 | 3.782 |
| **6** | **2** | Youth can't sit still, is restless or hyperactive | 1.238 | 0.079 | 1.692 | 1.971 |  | 1.862 | 0.226 | 1.617 | 3.199 |  | 1.922 | 0.985 | 1.958 | 2.957 |
| **7** | **3** | Youth is impulsive or acts without thinking | 1.677 | 0.370 | 2.439 | 3.077 |  | 2.811 | 0.601 | 2.209 | 5.425 |  | 3.713 | 0.646 | 1.690 | 6.939 |
| **13** | **4** | Youth gets in many fights | 1.750 | 1.633 | 3.249 | 3.053 |  | 2.301 | 1.722 | 2.832 | 3.910 |  | 1.766 | 1.984 | 3.648 | 3.095 |
| **15** | **5** | Youth lies or cheats | 1.666 | 0.823 | 3.053 | 3.093 |  | 1.807 | 0.751 | 3.137 | 3.454 |  | 1.987 | 0.713 | 2.438 | 3.614 |
| **16** | **6** | Youth physically attacks people | 2.754 | 1.672 | 3.164 | 5.222 |  | 2.199 | 2.121 | 3.372 | 3.804 |  | 2.123 | 2.316 | 4.190 | 3.942 |
| **22** | **7** | Youth threatens people | 1.821 | 1.800 | 3.079 | 3.011 |  | 2.086 | 2.194 | 3.453 | 3.561 |  | 2.780 | 1.844 | 2.611 | 4.430 |
| **23** | **8** | Youth vandalizes | 1.353 | 2.477 | 4.114 | 2.211 |  | 2.863 | 2.522 | 3.683 | 5.084 |  | 2.396 | 2.387 | 3.330 | 3.909 |
| **31** | **9** | Youth is disobedient at home | 2.019 | -0.441 | 1.932 | 3.909 |  | 2.194 | 0.124 | 2.388 | 4.270 |  | 1.987 | 0.434 | 2.245 | 3.673 |
| **32** | **10** | Youth is disobedient at school | 1.598 | 0.820 | 2.761 | 2.855 |  | 2.178 | 0.671 | 2.466 | 4.087 |  | 2.166 | 0.895 | 2.516 | 3.974 |

First-to-second eigenvalue ratio = 4.14–6.29 across T1–T3, exceeding the recommended 3:1 threshold; first eigenvalue explained 49–62% of variance; C_2_ statistic fit indices: CFI = 0.932–0.962, TLI = 0.918–0.959, RMSEA = 0.055–0.075; Yen's Q3 mean residual correlation = -0.05 to -0.08, with 3–7 item pairs exceeding the |Q3| > 0.20 threshold across timepoints.

Abbreviations: *IRT* Item Response Theory, *IP NO.* item pool number, *Cur NO.* original item number, *Info* amount of information between -4 SD and +4 SD around the latent trait mean, *T1–T3* timepoints 1–3, *a* discrimination/slope parameter, *b_1_–b_2_* difficulty parameters.

**eTable 7.** Various structural solutions and item versions of the CBCL.

| **Set Name** | **Solution** | **Items** |
| --- | --- | --- |
| Original | One-factor model for 34 items | G: 1-34 |
| Original | Five-factor model for 34 items | Anxiety Problems: 1-4  Attention Deficit/Hyperactivity Problems: 5-9  Conduct Problems: 10-23  Depressive Problems: 24-29  Oppositional Defiant Problems: 30-34 |
| FFCWS | Seven-factor model for 34 items | Social Problems: 1  Anxious/Depressed: 2, 3, 4, 24, 25, 26  Attention Problems: 5, 6, 7  Aggressive: 8, 9, 10, 11, 13, 16, 22, 30, 31, 32, 33, 34  Rule-breaking, behavior: 12, 14, 15, 17, 18, 19, 20, 21, 23  Thought Problems: 27  Withdrawn: 28, 29 |
| EGA T1 | Four-factor model for 34 items | F1: 1, 2, 3, 5, 6, 12, 24, 25, 27, 28, 29, 33, 34  F2: 4, 26  F3: 7, 8, 9, 15, 30, 31, 32  F4: 10, 11, 13, 14, 16, 17, 18, 19, 20, 21, 22, 23 |
| EGA T2 | Three-factor model for 34 items | F1: 1, 2, 3, 4, 24, 25, 26, 27, 28, 29  F2: 5, 6, 7, 8, 9, 14, 15, 30, 31, 32, 33, 34  F3: 10, 11, 12, 13, 16, 17, 18, 19, 20, 21, 22, 23 |
| EGA T3 | Five-factor model for 34 items | F1: 1, 2, 3, 4, 24, 25, 26, 27, 28, 29  F2: 5, 6, 7, 12  F3: 8, 9, 30, 33, 34  F4: 10, 11, 13, 16, 17, 18, 19, 20, 22, 23  F5: 14, 15, 21, 31, 32 |
| After reducing 24 items | One-factor model for 10 items | G: 5, 6, 7, 13, 15, 16, 22, 23, 31, 32 |
| Original after reducing 24 items | Three-factor model for 10 items | Attention Deficit/Hyperactivity Problems: 5, 6, 7  Conduct Problems: 13, 15, 16, 22, 23  Oppositional Defiant Problems: 31, 32 |
| FFCWS after reducing 24 items | Three-factor model for 10 items | Attention Problems: 5, 6, 7  Aggressive: 13, 16, 22, 31, 32  Rule-breaking behavior: 15, 23 |
| EGA T1 after reducing 24 items | Three-factor model for 10 items | F1: 5, 6  F2: 13, 16, 22, 23  F3: 7, 15, 31, 32 |
| EGA T2&T3 after reducing 24 items | Three-factor model for 10 items | F1: 5, 6, 7  F2: 13, 16, 22, 23  F3: 15, 31, 32 |

Item number marked refers to the original item pool number.

Abbreviations: *FFCWS* The Future of Families and Child Wellbeing Study, *EGA* Exploratory Graph Analysis.

**eTable 8.** Goodness-of-fit indices of different CBCL models (Confirmatory data, *N* = 893).

| **Model** | ***χ^2^*** | ***df*** | **CFI** | **TLI** | **RMSEA (90% CI)** |
| --- | --- | --- | --- | --- | --- |
| Full 1F Original T1 | 1457.65 | 527 | 0.863 | 0.854 | 0.044 (0.042, 0.047) |
| Full 5F Original T1 | 2494.84 | 526 | 0.710 | 0.691 | 0.065 (0.062, 0.067) |
| Full 7F FFCWS T1 | 1226.93 | 509 | 0.894 | 0.884 | 0.040 (0.037, 0.043) |
| Full 4F EGA1 T1 | 948.472 | 521 | 0.937 | 0.932 | 0.030 (0.027, 0.033) |
| Full 3F EGA2 T1 | 1225.97 | 524 | 0.897 | 0.889 | 0.039 (0.036, 0.042) |
| Full 5F EGA3 T1 | 1072.69 | 517 | 0.918 | 0.911 | 0.035 (0.032, 0.038) |
| 10-item 1F Original T1 | 377.15 | 35 | 0.857 | 0.816 | 0.105 (0.095, 0.114) |
| 10-item 3F Original T1 | 192.304 | 32 | 0.933 | 0.906 | 0.075 (0.065, 0.085) |
| 10-item 3F FFCWS T1 | 304.038 | 33 | 0.887 | 0.845 | 0.096 (0.086, 0.106) |
| 10-item 3F EGA1 T1 | 84.302 | 32 | 0.978 | 0.969 | 0.043 (0.032, 0.054) |
| 10-item 3F EGA2/3 T1 | 196.509 | 32 | 0.931 | 0.903 | 0.076 (0.066, 0.086) |
| Full 1F Original T2 | 377.15 | 35 | 0.857 | 0.816 | 0.105 (0.095, 0.114) |
| Full 5F Original T2 | 5864.36 | 526 | 0.599 | 0.572 | 0.107 (0.104, 0.109) |
| Full 7F FFCWS T2 | 1117.52 | 509 | 0.954 | 0.950 | 0.037 (0.034, 0.040) |
| Full 4F EGA1 T2 | 1354.83 | 521 | 0.937 | 0.932 | 0.042 (0.040, 0.045) |
| Full 3F EGA2 T2 | 1171.43 | 524 | 0.951 | 0.948 | 0.037 (0.034, 0.040) |
| Full 5F EGA3 T2 | 1067.94 | 517 | 0.959 | 0.955 | 0.035 (0.032, 0.037) |
| 10-item 1F Original T2 | 286.467 | 35 | 0.943 | 0.926 | 0.090 (0.080, 0.100) |
| 10-item 3F Original T2 | 128.053 | 32 | 0.978 | 0.969 | 0.058 (0.048, 0.069) |
| 10-item 3F FFCWS T2 | 180.309 | 33 | 0.966 | 0.954 | 0.071 (0.061, 0.081) |
| 10-item 3F EGA1 T2 | 129.385 | 32 | 0.978 | 0.969 | 0.058 (0.048, 0.069) |
| 10-item 3F EGA2/3 T2 | 135.581 | 32 | 0.976 | 0.967 | 0.060 (0.050, 0.071) |
| Full 1F Original T3 | 2160 | 527 | 0.899 | 0.893 | 0.059 (0.056, 0.062) |
| Full 5F Original T3 | 4838.54 | 526 | 0.734 | 0.716 | 0.096 (0.093, 0.098) |
| Full 7F FFCWS T3 | 1356.72 | 509 | 0.948 | 0.942 | 0.043 (0.040, 0.046) |
| Full 4F EGA1 T3 | 1760.03 | 521 | 0.923 | 0.918 | 0.052 (0.049, 0.054) |
| Full 3F EGA2 T3 | 1307.54 | 524 | 0.952 | 0.948 | 0.041 (0.038, 0.044) |
| Full 5F EGA3 T3 | 1109.99 | 517 | 0.963 | 0.960 | 0.036 (0.033, 0.039) |
| 10-item 1F Original T3 | 269.982 | 35 | 0.959 | 0.947 | 0.087 (0.077, 0.097) |
| 10-item 3F Original T3 | 100.246 | 32 | 0.988 | 0.983 | 0.049 (0.038, 0.060) |
| 10-item 3F FFCWS T3 | 95.7 | 33 | 0.989 | 0.985 | 0.046 (0.036, 0.057) |
| 10-item 3F EGA1 T3 | 161.379 | 32 | 0.977 | 0.968 | 0.067 (0.057, 0.078) |
| 10-item 3F EGA2/3 T3 | 87.978 | 32 | 0.990 | 0.986 | 0.044 (0.033, 0.055) |

Abbreviations: *χ^2^* Chi-square, *df* degrees of freedom, *CFI* comparative fit index, *TLI* Tucker-Lewis index, *RMSEA* root mean square error of approximation, *CI* confidence interval.

**eTable 9.** IRT parameters of the final 10-item version at T1, T2, and T3 (Full set, *N* = 1786).

| **IP NO.** | **Cur NO.** | **Content** | **T1** | | | |  | **T2** | | | |  | **T3** | | | |
| --- | --- | --- | --- | --- | --- | --- | --- | --- | --- | --- | --- | --- | --- | --- | --- | --- |
|  |  |  | **a** | **b_1_** | **b_2_** | **Info** |  | **a** | **b_1_** | **b_2_** | **Info** |  | **a** | **b_1_** | **b_2_** | **Info** |
| **5** | **1** | Youth can't concentrate, can't pay attention for long | 1.156 | 0.435 | 2.678 | 1.975 |  | 1.782 | 0.119 | 1.768 | 2.252 |  | 2.273 | 0.433 | 1.751 | 3.993 |
| **6** | **2** | Youth can't sit still, is restless or hyperactive | 1.144 | 0.121 | 1.901 | 1.826 |  | 1.756 | 0.287 | 1.691 | 2.336 |  | 2.073 | 0.956 | 1.999 | 3.359 |
| **7** | **3** | Youth is impulsive or acts without thinking | 1.606 | 0.442 | 2.485 | 2.908 |  | 2.574 | 0.587 | 2.336 | 3.450 |  | 3.139 | 0.605 | 1.819 | 5.834 |
| **13** | **4** | Youth gets in many fights | 2.044 | 1.578 | 2.991 | 3.583 |  | 2.144 | 1.781 | 3.012 | 2.428 |  | 2.016 | 1.817 | 3.080 | 3.405 |
| **15** | **5** | Youth lies or cheats | 1.434 | 0.907 | 3.412 | 2.645 |  | 1.850 | 0.721 | 3.064 | 2.467 |  | 1.978 | 0.685 | 2.530 | 3.654 |
| **16** | **6** | Youth physically attacks people | 2.472 | 1.747 | 3.178 | 4.542 |  | 2.356 | 2.038 | 3.126 | 3.232 |  | 2.258 | 2.222 | 3.736 | 4.069 |
| **22** | **7** | Youth threatens people | 1.733 | 1.932 | 3.334 | 2.904 |  | 2.517 | 2.018 | 3.090 | 3.873 |  | 2.238 | 1.916 | 2.961 | 3.685 |
| **23** | **8** | Youth vandalizes | 1.434 | 2.460 | 3.875 | 2.283 |  | 2.914 | 2.570 | 3.390 | 6.063 |  | 1.899 | 2.654 | 3.993 | 3.184 |
| **31** | **9** | Youth is disobedient at home | 1.896 | -0.443 | 2.013 | 3.653 |  | 1.943 | 0.078 | 2.531 | 2.638 |  | 1.958 | 0.403 | 2.290 | 3.635 |
| **32** | **10** | Youth is disobedient at school | 1.535 | 0.862 | 2.935 | 2.756 |  | 2.222 | 0.647 | 2.559 | 2.728 |  | 2.347 | 0.827 | 2.291 | 4.301 |

First-to-second eigenvalue ratio = 4.03–6.39 across T1–T3; C_2_ statistic fit indices: CFI = 0.922–0.950, RMSEA = 0.084–0.093; Yen's Q3 mean residual correlation = -0.081 to -0.085, with 2–7 adjusted item pairs exceeding the |Q3 − mean| > 0.20 threshold across timepoints.

Abbreviations: *IRT* Item Response Theory, *IP NO.* item pool number, *Cur NO.* original item number, *Info* amount of information between -4 SD and +4 SD around the latent trait mean, *T1–T3* timepoints 1–3, *a* discrimination/slope parameter, *b_1_–b_2_* difficulty parameters.

**eTable 10.** Internal consistency of the revised 10-item CBCL (Full data, *N* = 1786).

| **Variables** | **CBCL** | **AP** | **TB** | **OD** |
| --- | --- | --- | --- | --- |
| **Cronbach’s α (95% CI)** | | | | |
| T1 | 0.880 (0.872, 0.889) | 0.732 (0.711, 0.754) | 0.816 (0.802, 0.830) | 0.776 (0.757, 0.794) |
| T2 | 0.934 (0.929, 0.939) | 0.861 (0.850, 0.873) | 0.911 (0.904, 0.918) | 0.838 (0.825, 0.852) |
| T3 | 0.932 (0.927, 0.937) | 0.907 (0.899, 0.914) | 0.852 (0.841, 0.863) | 0.835 (0.822, 0.848) |
| **McDonald’s ω (95% CI)** | | | | |
| T1 | 0.880 (0.871, 0.888) | 0.754 (0.735, 0.773) | 0.823 (0.810, 0.837) | 0.781 (0.764, 0.799) |
| T2 | 0.933 (0.928, 0.938) | 0.863 (0.852, 0.874) | 0.915 (0.909, 0.922) | 0.855 (0.844, 0.866) |
| T3 | 0.932 (0.928, 0.937) | 0.908 (0.900, 0.915) | 0.852 (0.841, 0.863) | 0.844 (0.832, 0.856) |

This table shows ordinal versions of Cronbach’s α and McDonald’s ω.

Abbreviations: *CBCL* Child Behavior Checklist, *AP* Attention Problems, *TB* Threatening Behaviour, *OD* Oppositional Defiant.
